# Targeted complement inhibition ameliorates the pathological and cognitive outcomes in repetitive mild closed head injury

**DOI:** 10.1038/s41392-025-02466-7

**Published:** 2025-11-20

**Authors:** Khalil Mallah, Carsten Krieg, Devin Hatchell, Nahla Hamouda, Tylar Roof, Stephen Walterhouse, Amer Toutonji, Davis Borucki, Christine Couch, Gary Hardiman, Firas Kobeissy, Silvia Guglietta, Stephen Tomlinson

**Affiliations:** 1 Department of Pharmacology and Immunology, Medical University of South Carolina, Charleston, SC USA; 2 Ralph H. Johnson Department of Veterans Affairs Medical Center, Charleston, SC USA; 3 Department of Pathology and Laboratory Medicine, Medical University of South Carolina, Charleston, SC USA; 4 Department of Dermatology, Medical University of South Carolina, Charleston, SC USA; 5 Department of Regenerative Medicine & Cell Biology, Medical University of South Carolina, Charleston, SC USA; 6 Department of Clinical Pharmacology, Faculty of Medicine, Alexandria University, Alexandria, Egypt; 7 Department of Neurosciences, Medical University of South Carolina, Charleston, SC USA; 8 Department of Health Sciences and Research, College of Health Professions, Medical University of South Carolina, Charleston, SC USA; 9 Institute for Global Food Security, School of Biological Sciences, Queens University Belfast, Belfast, UK; 10 Department of Biochemistry and Molecular Genetics, Faculty of Medicine, American University of Beirut, Beirut, Lebanon; 11 Department of Neurobiology, Center for Neurotrauma, Multiomics & Biomarkers, Morehouse School of Medicine, Neuroscience Institute, Atlanta, GA USA; 12 Hollings Cancer Center, Charleston, SC USA

**Keywords:** Neuroimmunology, Inflammation

## Abstract

Repeated mild closed head injury (rmCHI) is a significant public health concern, and this type of repetitive injury is garnering increasing attention, not least because of its increasing incidence in sports. The underlying neuroimmune mechanisms secondary to trauma that link rmCHI to cognitive impairment remain to be elucidated, and the contribution of the complement system to the pathological sequelae of this type of brain injury is unexplored. Here, using C57BL/6J mice, we established a repetitive 12-head impact model to investigate the neuroimmune and pathological processes that occur after rmCHI. We specifically studied the role of complement in pathology and cognitive impairment up to 21 days after the cessation of injury in a clinically relevant paradigm using the site-targeted complement inhibitor CR2-Crry. Our analytical methods included mass cytometry, RNA-seq, proteomics, and immunohistological characterization. Mass cytometric analysis revealed that cognitive impairment after rmCHI was associated with major subacute/chronic alterations in local immune cell recruitment, particularly the recruitment and activation of microglia, with marked upregulation of complement receptors and proteins associated with the phagocytic machinery. RNA-seq and proteomic analysis revealed major changes in pathways associated with neurodegeneration, neuronal apoptosis, and the upregulation of complement proteins in animals subjected to rmCHI. Complement inhibition initiated after cessation of injury modulated rmCHI-induced changes and protected against cognitive impairment. In addition to expanding our understanding of the pathological sequelae of rmCHI, these data highlight the therapeutic potential of complement inhibition.

## Introduction

Traumatic brain injury (TBI) is a consequence of a physical impact to the head, which leads to an alteration in brain function and mental state. There are three main TBI categories based on the physical mechanism of damage: a) closed head, b) penetrating (open head), and c) explosive blast-induced TBI.^1^ In recent years, repetitive closed-head TBI has become a focus of TBI-related research because of its increasing incidence rate, particularly in the context of contact sports. Concussions are the most common type of mild TBI,^2^ and a study that included 13,088 adolescents surveyed in 2016 revealed that 19.5% of these individuals were diagnosed with at least one concussion in their lifetime.^3^ Also in the same study, 5.5% of the surveyed individuals reported a diagnosis of more than one concussion. TBI poses a significant challenge to modern healthcare due to its extensive physical, psychiatric, and cognitive consequences, which profoundly impact not only the individuals affected but also their families.^4^ In addition to its human toll, TBI imposes a substantial financial burden, with global economic costs estimated at approximately $400 billion annually. Recent studies have shown that individuals with TBI, both adults and children, are significantly more likely than those without TBI to require outpatient care, emergency department visits, and hospitalizations.^4,5^ Importantly, TBI is also a major risk factor for the development of several chronic neurological and psychiatric disorders, such as post-traumatic stress disorder (PTSD), Alzheimer’s disease (AD), amyotrophic lateral sclerosis (ALS), depression, suicide, epilepsy, stroke, and Parkinson’s disease.^6^ Within the framework of preclinical models, there is a well-established neuroimmune response following TBI, although somewhat less is known about this response in repetitive mild closed head injury (reviewed in ref. ^7^).

In TBI, the primary mechanical injury is followed by a secondary neuroinflammatory reaction that includes processes such as excitotoxicity, mitochondrial dysfunction, oxidative stress, demyelination, and subsequent neurodegeneration.^8^ An integral component of secondary neuroinflammation is the neuroimmune response, which initiates within seconds after injury and can persist for days, months, or possibly years, depending on injury severity.^9,10^ We, and others, have shown a role for the complement system in the secondary neuroimmune response following TBI,^11–18^ and preclinical experimental findings are supported by clinical data.^19,20^ The complement system is an important component of both innate and adaptive immunity and includes more than 50 circulating or cell-associated proteins.^21^ The complement system has numerous functions, including defense against pathogens, recruitment of immune cells and modulation of their activity, tissue repair and regeneration, and various homeostatic activities. These various activities are executed principally through the complement activation products C3a and C5a (anaphylatoxins), C3 opsonins and their recognition by complement receptors, and the cytolytic membrane attack complex (MAC).^22–24^ Notably, we previously reported that following moderate to severe injury by controlled cortical impact (CCI), C3 complement activation products are deposited on perilesional synapses, which results in their recognition and phagocytic removal by microglia.^25^ This aberrant process of complement-mediated phagocytosis of synapses occurs in both brain hemispheres (ipsilateral and contralateral) and is associated with an ongoing cognitive decline measured up to 6 months after injury.^16,25^ Complement inhibition after CCI halted cognitive decline and increased synapse density, even when treatment with a complement inhibitor was initiated as late as 2 months after injury.^16,25^

Preclinical investigations into the role of complement in closed-head TBI are somewhat limited. To our knowledge, no studies have examined the role of complement in repetitive mild closed head injury (rmCHI), a common form of TBI, particularly among contact sport athletes and military personnel. Previous preclinical studies examining the role of the complement system in closed head TBI utilized a model of single-hit, usually severe, injury (for reviews on complement and TBI, see refs. ^26,27^). To address this critical gap in knowledge, we developed a murine model of rmCHI to test the hypothesis that complement contributes to the pathogenesis of this form of head injury. Using a combination of multiomic approaches, we characterized the cellular and molecular changes associated with rmCHI. In parallel, we employed the site-targeted complement inhibitor CR2-Crry to study the role of complement in post-injury pathology within clinically relevant experimental paradigms.

CR2-Crry is a fusion protein consisting of the C3-recognizing domain of complement receptor 2 (CR2) and the complement inhibitor Crry. The CR2 domain serves as a targeting vehicle that localizes the construct to sites of complement activation (C3 deposition), and the Crry domain is an inhibitor of all complement pathways by preventing the activation of C3. The CR2-Crry inhibitor is neuroprotective in several murine CNS injury models, including stroke,^28^ spinal cord injury^29^ and CCI.^14,16,25^

## Results

### Optimization of a murine model of repetitive mild closed head injury

**Fig. 1:**
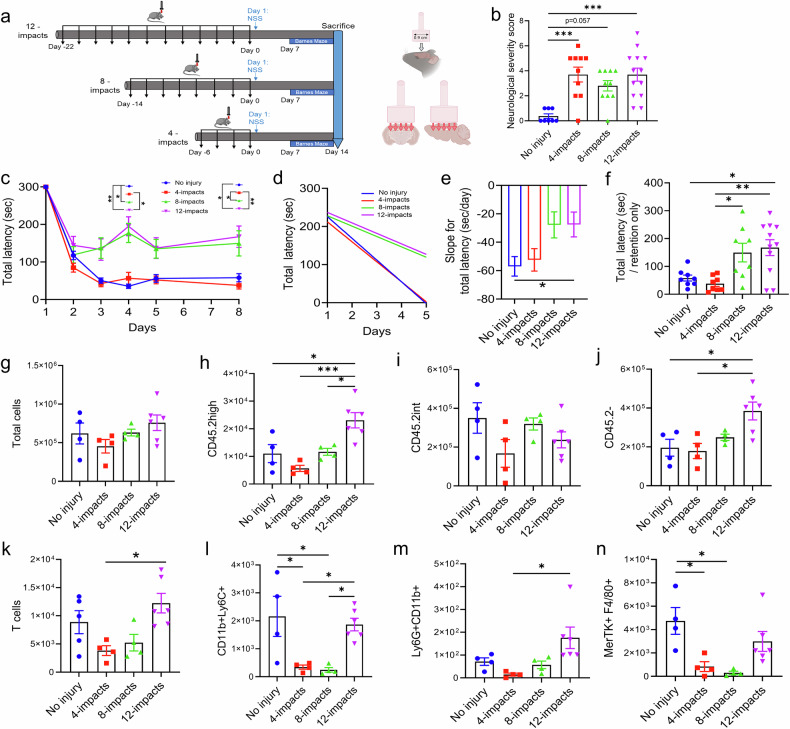
Optimization of a repetitive mild closed head injury (rmCHI) model of TBI in mice. **a** Experimental design showing the number and timing of closed head impacts. We also provide an illustration of the location of the impact and the underlying brain areas immediately involved. Created in BioRender.com **b** Neurological severity score (NSS) at 6 h after the last impact. Number of replicates: No injury (*n* = 8), 4 impacts (*n* = 10), 8 impacts (*n* = 10), and 12 impacts (*n* = 13). Statistical analysis was carried out via the Kruskal‒Wallis test with Dunn’s test for multiple comparisons of each condition. Medians are shown, *** =*p* < 0.001. **c** Spatial learning and memory retention were assessed via the Barnes Maze as indicated by the total latency to escape the platform. This task started on day 7 after the last impact. Statistical analysis was carried out via repeated-measures two-way ANOVA with the Bonferroni correction for multiple comparisons. Mean +/− SEM. * =*p* < 0.05; ** =*p* < 0.01 **d** Learning phase for the first 5 days of the Barnes maze test. **e** Slope of improvement in total latency for the first 5 days of the task. Statistical analysis was carried out via one-way ANOVA with the Bonferroni correction for multiple comparisons. An unpaired t-test was also performed to compare non-injured and 12-impact mice. Mean +/− SEM. *p < 0.05. **f** Comparison of memory retention on day 8 of the task by measuring the total latency (sec) to escape the platform. Statistical analysis was carried out via one-way ANOVA with Bonferroni correction for multiple comparisons. An unpaired t test was also performed to compare non-injured and 12-impact mice. Mean +/− SEM. *p < 0.05; **p < 0.01. For **c**–**f**, the number of replicates: no injury (*n* = 8), 4 impacts (*n* = 8), 8 impacts (*n* = 8), and 12 impacts (*n* = 11). **g** Bar graphs showing the total number of cells in the brains of non**-**injured mice and rmCHI mice after 4, 8, and 12 impacts at 14 days after the last impact. **h** CD45.2^high^ immune cells. **i** CD45.2^int^ immune cells. **j** CD45.2^-^ nonimmune cells. **k** T cells**. l** Monocytes. **m** neutrophils and **n** Macrophages. A minimum of 4 animals/group were used for flow cytometry. For **g**‒**n**, the analysis was performed via one-way ANOVA with the Bonferroni correction for multiple comparisons. Mean +/- SEM. *= *p* < 0.05; **= *p* < 0.01; ***= *p* < 0.001

The study design for initial model development consisted of 4 experimental groups with mice receiving 0, 4, 8, or 12 mild closed head impacts. The time between each impact was 48 hours, and for model development, all animals were euthanized 14 days after the final impact for characterization of the outcome (refer to the schematic in Fig. 1a).

To assess short-term motor and sensory deficits, the neurological severity score (NSS) was determined at 6 h after the final impact. Compared with age-matched non-injured controls, all three injury groups (4, 8, and 12 impacts) exhibited a worse NSS (Fig. 1b). There was no difference in NSS among the three injury groups. We also assessed NSS at later time points and found that NSS returned to normal by 24 h after the last impact (Supplementary Fig. 1). This finding is reminiscent of our previous data from a CCI model of more severe TBI in which the NSS returned to baseline by 5 days after injury.^30^ These data are also a general reflection of clinical findings that usually show only short-term motor and sensory deficits after mild repetitive brain injury.^31^ To assess cognitive performance, we performed the Barnes Maze task to measure spatial learning and memory retention. The task started on day 7 after the last impact for each of the three groups. Compared with the 8- and 12-impact groups, during the spatial learning phase (first 5 days), the non-injured and 4-impact groups presented similar and improved spatial learning performance (Fig. 1c). The learning slope (total latency vs. time) was significantly steeper for non-injured vs. 12-impact animals, and there was no difference between non-injured and 4-impact animals (Fig. 1d, e). There was a trend toward worse outcomes for the 8-impact animals compared with non-injured animals, but the difference did not reach significance. These results were replicated with regard to memory retention measured on day 8 of the task, in which only the 12-impact group showed a significantly increased latency to enter the escape hole compared with the non-injured and 4-impact groups (Fig. 1f).

TBI is associated with the recruitment and activation of immune cells that can promote a neuroinflammatory process, leading to secondary injury and long-term pathologic sequalae.^32^ We therefore investigated how the different injury paradigms affected the number and phenotype of immune cells in the brain. The gating strategy for the identification of the different cell populations is detailed in Supplementary Fig. 2. In general, the 12-impact group presented a significantly greater number of immune cells, defined as CD45.2^high^ cells, than the non-injured and the 4- and 8-impact groups did (Fig. 1h). Among these immune cells, neutrophils (Ly6G^+^CD11b^+^) and T cells, but not monocytes (CD11b^+^Ly6C^+^) or macrophages (MerTK+F4/80 + ), were the prominent cell types accounting for the difference in the number of CD45.2high cells in the 12 impact group (Fig. 1k–n). Conversely, there was no significant difference in the number of CD11b^+^ CD45.2^int^ cells, identified as resident mononuclear phagocytes (microglia and border-associated macrophages), between the three different injury paradigms and non-injured animals (Fig. 1i). Interestingly, the number of nonimmune cells (CD45.2-) was significantly greater in the 12-impact group than in the non-injured and 4-impact groups (Fig. 1j). Notably, the changes observed in both immune and nonimmune cells in the brain were due to repetitive impacts and not repetitive injections of anesthesia; we found no difference in the number of immune or nonimmune cells between non-injured animals that received 12 doses of anesthesia and non-injured animals that received no anesthesia (Supplementary Fig. 3).

In summary, the above data show that both the 8- and 12-impact injury paradigms resulted in behavioral deficits compared with the non-injured group, but 12-impact injury resulted in the most significant alterations in immune cell profiles. Thus, subsequent investigations into the role of complement in rmCHI were performed using the 12-impact injury model.

### Repetitive mild closed head injury results in the activation of microglia and complement

**Fig. 2:**
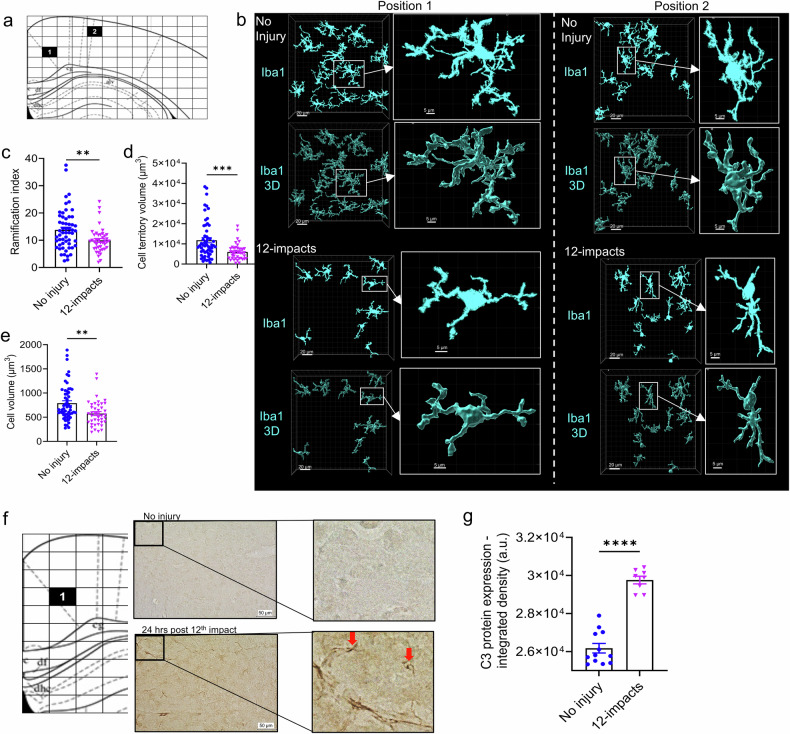
rmCHI induces morphological changes in microglia in the cortex as well as increased C3 activation. **a** Atlas image showing the location of the images captured via the confocal microscope (bregma −1.70 mm). **b** Representative 63x images of microglia (Iba1) in the cortex of non-injured mice and 12-impact rmCHI mice 14 days after the last hit. **c** Ramification index obtained from the 3D morph analysis. **d** Cell territory volume obtained from the 3D morph analysis. **e** Cell volume obtained from the 3D morph analysis. For **c**–**e**, number of replicates: no injury (*n* = 56 cells combined from 3 animals and 2 images per animal) and 12 impacts (*n* = 41 cells combined from 3 animals and 2 images per animal). Statistical analysis was performed via the Mann‒Whitney test. Mean +/− SEM. **=*p* < 0.01; ***=*p* < 0.001. **f** Brain Atlas image showing the location of the images captured via confocal microscopy (bregma–1.70 mm), along with representative 40x IHC images of C3 staining in non-injured and 24 h post the 12th impact in the rmCHI group. Red arrows indicate areas of increased complement deposition. **g** Quantification of C3 deposition in non-injured mice vs 24 h post last impact. A total of *n* = 3 animals per condition and 2–4 images per animal were acquired. Statistical analysis was performed via the Mann‒Whitney test. Mean +/− SEM. ****= *p* < 0.0001

Microglial morphology can be used as a phenotypic indicator of activation status, and we assessed injury-induced alterations in microglial morphology in the brain 14 days after 12-impact injury. In general, an increase in microglial ramification is indicative of a resting/patrolling phenotype, whereas a decrease in microglial ramification and a more ameboid morphology is associated with a more activated phenotype. Microglial morphology was analyzed via confocal microscopy of cortical brain regions from non-injured mice and from mice subjected to the 12-impact injury paradigm (Fig. 2a, b). Cell morphology parameters (including ramification parameters) were obtained via the 3D morph script, which allows semi-automatic processing of individual cells. Compared with microglia in non-injured brains, microglia in rmCHI brains presented a significantly lower ramification index, lower cell territory volume, and lower cell volume (Fig. 2c–e) (Supplementary Movie 1–4). These data show that rmCHI induces a less ramified microglial state, indicating a more activated state. Our prior work has shown that complement plays a central role in the neuroinflammatory process that occurs after severe contusive TBI (CCI) in both the acute and chronic phases after injury^14–16,25^; however, the role of complement in rmCHI or concussive TBI is largely unexplored. To this end, we demonstrated complement activation in the brain after rmCHI, as evidenced by C3 deposition in the cortical tissue of the brains of the rmCHI mice at 24 h after the last (12th) impact (Fig. 2f, g).

### Complement inhibition improves cognitive performance after rmCHI

**Fig. 3:**
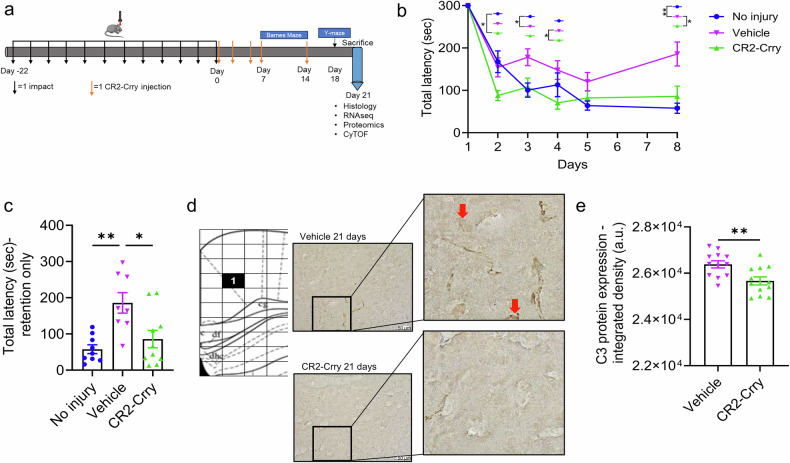
Complement inhibition after rmCHI improved cognitive performance. **a** Experimental design showing the impact and CR2-Crry treatment schedule. **b** Spatial learning and memory retention were assessed via the Barnes Maze as indicated by the total latency to escape the platform. Statistical analysis for **b** was carried out via repeated-measures two-way ANOVA with Bonferroni correction for multiple comparisons. Mean +/− SEM. * =*p* < 0.05; ** =*p* < 0.01. **c** Comparison of memory retention on day 8 of the Barnes Maze test was performed by measuring the total latency to escape the platform. Statistical analysis for **c** was carried out via one-way ANOVA with the Bonferroni correction for multiple comparisons. Mean +/− SEM. * =*p* < 0.05; ** =*p* < 0.01. For **b**, **c**, the number of replicates was as follows: no injury (*n* = 9), vehicle (*n* = 8), and CR2-Crry (*n* = 10). **d** Atlas image showing the location of the images captured via confocal microscopy (bregma −1.70 mm) along with representative 40x IHC images showing C3 deposition in vehicle- vs. CR2-Crry-treated animals. **e** Quantification of C3 deposition in the cortex of vehicle- vs. CR2-Crry-treated animals measured 21 days after the last (12th) impact. Number of replicates: Vehicle (*n* = 12 images from 3 animals) and CR2-Crry (*n* = 12 images from 3 animals). Statistical analysis was performed via an unpaired t-test. Mean +/− SEM. **= *p* < 0.01

We more directly investigated the role of complement by using the complement inhibitor CR2-Crry, which binds to sites of complement activation and C3 deposition. Mice were treated with a total of five intraperitoneal (I.P.) injections of CR2-Crry starting after the last (12th) impact and continuing until the experimental endpoint at 21 days after the last impact (Fig. 3a). Specifically, the animals received I.P. injections on days 0, 2, 4, 7, and 14 after the last impact. Animals treated with CR2-Crry demonstrated improved cognitive performance in the Barnes maze task relative to their vehicle-treated counterparts (Fig. 3b). This was especially evident on the retention day, in which CR2-Crry-treated animals found the escape hole in a significantly shorter time than vehicle-treated animals at 21 days post last impact (Fig. 3c). Furthermore, improved cognitive performance correlated with reduced cortical deposition of C3 in CR2-Crry-treated vs. vehicle-treated animals (Fig. 3d, e).

### Complement inhibition after rmCHI results in the transcriptional upregulation of pathways associated with neuroprotection, synaptic plasticity, and neurogenesis

We performed transcriptomic analysis via bulk RNA-seq of brain tissue isolated from non-injured animals and from injured animals treated with either CR2-Crry or vehicle. Before brain isolation for analysis, we confirmed that the rmCHI cohorts exhibited cognitive deficits and that CR2-Crry treatment reduced complement activation within the brain. In agreement with the above data, vehicle-treated rmCHI mice performed worse on the Barnes maze task than non-injured and CR2-Crry-treated animals did; they also performed worse on the Y-maze test, which is a cognitive task that assesses spatial working memory (Supplementary Fig. 4a, b).

**Fig. 4:**
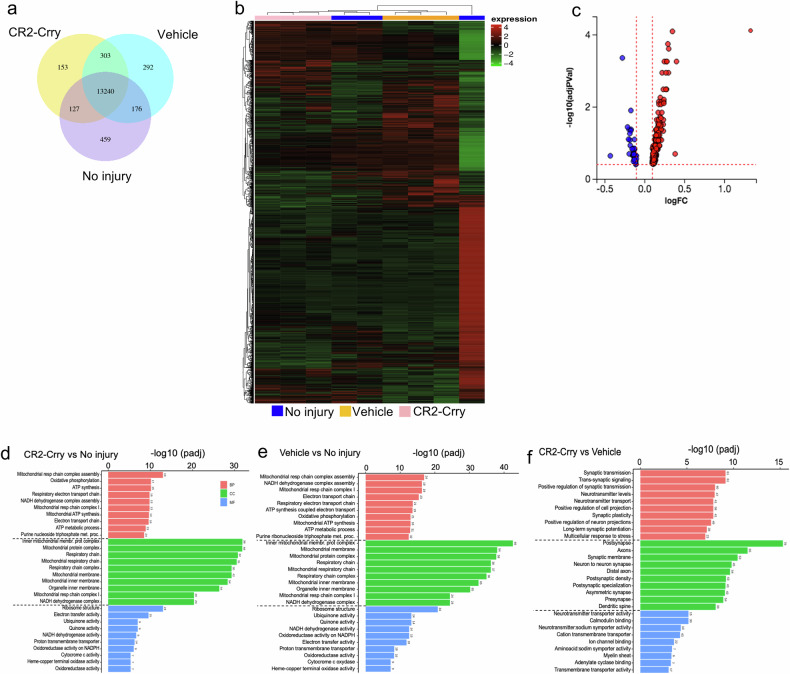
Complement inhibition after rmCHI results in the transcriptional upregulation of pathways associated with neuroprotection, synaptic plasticity, and neurogenesis. **a** Venn diagram showing the number of genes that are uniquely expressed by non-injured mice and in rmCHI mice treated with vehicle or CR2-Crry at 21 days after the last impact. **b** Heatmap showing the overall results of the FPKM cluster analysis. Red indicates genes with high expression levels, and blue indicates genes with low expression levels. The color bar on top shows the different experimental groups detailed in the legend. **c** Volcano plot illustrating the magnitude of the fold change in the expression of all genes whose expression was upregulated (red) or downregulated (blue) in the brains of CR2-Crry- or vehicle-treated mice at 21 days after the last impact (*n* = 3). The vertical and horizontal red dotted lines indicate the threshold. Significance was calculated via the DESeq2 package with Benjamini‒Hochberg correction for the false discovery rate (FDR). An FDR cutoff of 5% and a minimum fold change of 1.5 (log2FC = 0.6) were applied to determine differential expression. Visualization of the top 10 Gene Ontology (GO) terms in the three categories: biological process (BP), cellular component (CC) and molecular function (MF) in the brains of CR2-Crry-treated vs no injury (**d**), vehicle vs no injury (**e**) and CR2-Crry-treated vs vehicle (**f**). The numbers on top of the bars indicate the number of genes in each pathway. The significance of enrichment on the x-axis is expressed as -log10(padj)

As shown in the Venn diagram in Fig. 4a, 13,240 genes were co-expressed in all three cohorts, with 153 genes uniquely expressed in CR2-Crry-treated animals, 292 genes uniquely expressed in vehicle-treated animals, and 459 genes uniquely expressed in non-injured animals. By using mainstream hierarchical clustering, we clustered the fragments per kilobase of transcript per million mapped reads (FPKM) values of genes and homogenized the row (Z-score) to generate a heatmap. As shown in Fig. 4b, CR2-Crry-treated animals exhibited a gene expression profile more closely resembling that of non-injured animals than that of vehicle-treated animals; thus, in the heatmap, CR2-Crry clustered closely with the non-injured group. By applying a *p*-adjusted value < 0.05, we identified 324 genes that were differentially expressed between the vehicle-treated group and the CR2-Crry-treated group at a false discovery rate (FDR) of 0.4 (Fig. 4c). The functional enrichment analysis of differentially expressed genes (DEGs) was performed with iPathway software via the Gene Ontology (GO) resource. As previously described, GO annotates genes to biological processes (BP), molecular functions (MF), and cellular components (CC).^33^ Figure 4d–f shows the top 10 functional enrichments in BP, MF and CC in CR2-Crry vs. No Injury (Fig. 4d), Vehicle vs. No Injury (Fig. 4e) and CR2-Crry vs. Vehicle (Fig. 4f). As detailed in panels d and e, compared with the non-injured group, both injured groups exhibited upregulated expression of genes associated with mitochondrial function, cellular respiration, and electron transport. However, relative to vehicle treatment, CR2-Crry treatment was associated with the upregulation of genes involved in neuron development, neurogenesis, memory, and synapse transmission (Fig. 4f). Overall, these data indicate that complement inhibition mitigates cognitive impairment and promotes transcriptional programs that preserve neuronal and synaptic integrity.

### Complement activation modifies the immune cell landscape of the injured brain after rmCHI

**Fig. 5:**
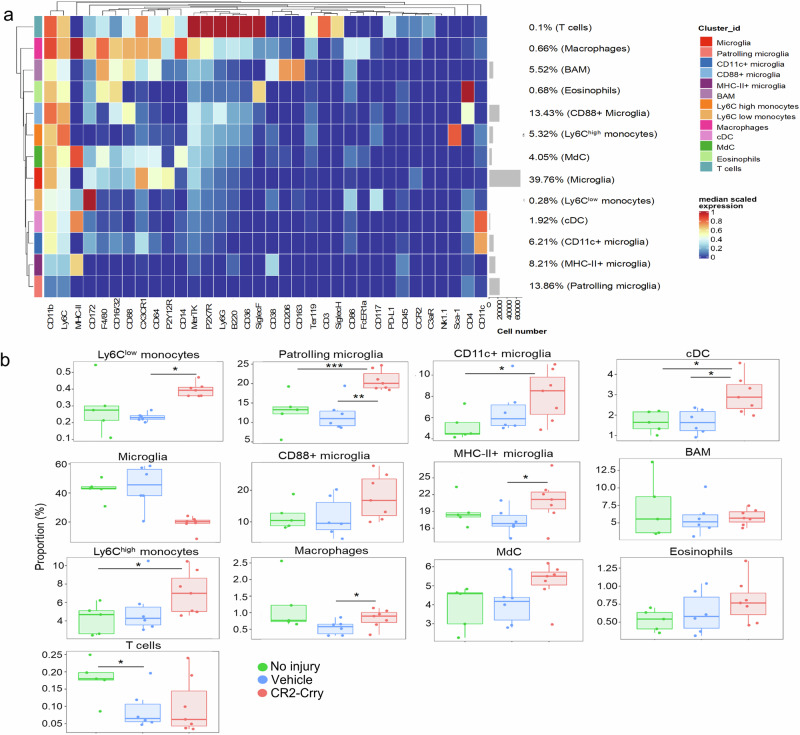
Complement inhibition modifies the immune cell landscape of the injured brain after rmCHI. **a** Heatmap showing lineage marker expression and abundance of the 13 immune cell types identified by mass cytometry in the brains of rmCHI mice at 21 days after the last impact. The side bars show the color code for each cell population and the median scaled expression of the lineage markers by each cell population. **b** Boxplots showing the percentage of the 13 immune cell types in vehicle- and CR2-Crry-treated rmCHI mice. A minimum of 5 mice/group were used for mass cytometry analysis. The false discovery rate was used to adjust the *p*-value. (*= *p* < 0.05; **= *p* < 0.01; ***= *p* < 0.001; ****= *p* < 0.0001)

We prepared single-cell suspensions from the brains of non-injured animals and from the brains of rmCHI animals treated with either CR2-Crry or vehicle and analyzed the cell populations via mass cytometry. The samples were barcoded using anti-CD45 following the barcoding scheme that we previously published,^30^ pooled and then stained with a cocktail of 34 antibodies. Data from at least of 1 × 10^6^ cells/batch were acquired, debarcoded, and analyzed as summarized in the workflow in Supplementary Fig. 5. The distribution of the samples belonging to the three groups via MDS plots is shown in Supplementary Fig. 6a. Using the FlowSOM algorithm, based on the differential expression of the lineage markers included in our CyTOF panel, we identified 30 clusters. These 30 clusters were manually annotated and merged into 13 cell clusters using lineage marker expression, as shown in the heatmap in Fig. 5a. We then used 1000 single cells from each of the 18 experimental samples to generate a UMAP to visualize the distribution of the identified immune cells (Supplementary Fig. 6b).

The identified cell types included T cells, eosinophils, monocyte-derived cells (MdC), macrophages, border-associated macrophages (BAM), monocytes characterized by low and high Ly6C expression, conventional dendritic cells (cDCs), and notably, five distinct microglial subpopulations (Fig. 5b). A population of macrophages expressing high levels of CD14, MHC-II, F4/80 and CD88 but low levels of CD206 and therefore distinct from brain resident border-associated macrophages (BAM), was observed at a greater frequency in non-injured and CR2-Crry-treated rmCHI mice relative to vehicle-treated rmCHI mice. This increase was statistically significant in the CR2-Crry-treated mice compared with the vehicle rmCHI mice. Immunofluorescence staining of macrophages in the hippocampus confirmed the CyTOF-based findings for this cell population in the same cohort of mice (Supplementary Fig. 7a–c). Differential analysis revealed an increase in the proportion of Ly6C^low^ and Ly6C^high^ monocytes in CR2-Crry-treated rmCHI mice compared with vehicle-treated and non-injured mice, respectively. There was also an increased proportion of classic dendritic cells (cDC) in the CR2-Crry-treated rmCHI mice compared with the non-injured and vehicle-treated mice (Fig. 5b). Furthermore, among the 5 microglial subpopulations identified, 3 were significantly different among the three experimental groups. Specifically, microglia from CR2-Crry-treated mice exhibited increased levels of MHC-II+ microglia and CD11c+ microglia compared with those from vehicle-treated rmCHI mice and non-injured mice, respectively. Furthermore, CR2-Crry–treated animals exhibited increased numbers of resting/patrolling microglia compared with both non-injured and vehicle-treated rmCHI mice. As shown by its position on the UMAP (Supplementary Fig. 6b), this resting/patrolling microglia population is spatially proximate to CD11c⁺ microglia, suggesting close ontogenetic relatedness; however, compared with other microglial clusters, it exhibits low expression of lineage-defining and inflammatory markers. Immunofluorescence staining of hippocampal microglia revealed significantly increased microglial numbers in CR2-Crry-treated animals compared with vehicle-treated and non-injured animals (Supplementary Fig. 7a, b). Interestingly, compared with the rmCHI mice, the non-injured mice exhibited a significantly greater frequency of T cells in the brain. To further characterize the T cell compartment in the context of rmCHI, we analyzed the distribution of naïve, central memory, and effector memory T cell subsets, which are well defined in the murine brain (Supplementary Fig. 8a–c).^34^ Compared with vehicle-treated animals, both non-injured and CR2-Crry-treated mice exhibited significantly greater proportions of CD4⁺ T cells (Supplementary Fig. 8b). While there were no significant differences in the frequencies of naïve (CD62L⁺CD44⁻) or central memory (CD62L⁺CD44⁺) CD4⁺ T cells among the groups, CR2-Crry-treated mice exhibited a modest but significant increase in effector memory CD4⁺ T cells (CD62L⁻CD44⁺). In contrast, vehicle-treated rmCHI mice exhibited significant expansion of naïve CD8⁺ T cells (Supplementary Fig. 8c).

Next, we studied the impact of CR2-Crry treatment on the expression of the complement receptors CR3 and C5aR1. To this end, we performed immunofluorescence staining for both receptors in the hippocampus of rmCHI mice treated with either CR2-Crry or vehicle (Supplementary Fig. 9). Compared with vehicle treatment, CR2-Crry treatment significantly increased the expression of both C5aR1 and CR3 (Supplementary Fig. 9a, b and e, f, respectively). Notably, the proportion of Iba-1+ cells expressing C5aR1 and CR3 was elevated in CR2-Crry-treated animals (Supplementary Fig. 9d and h, respectively). These findings align with our CyTOF analysis, which identified a CD88+ (C5aR1+) microglial cluster that was more abundant in CR2-Crry-treated animals, although it did not reach statistical significance, likely due to signal dilution from analyzing whole brain tissue, whereas the relevant population was primarily localized to the hippocampus.

Intriguingly, C5aR1 expression levels in the hippocampus were significantly inversely correlated with escape latency on the Barnes Maze retention test day (R² = 0.64, *P* = 0.05; Supplementary Fig. 9c), indicating that higher receptor expression was associated with improved cognitive performance. A similar inverse correlation was observed between CR3 expression and escape latency (R² = 0.68, *P* = 0.04; Supplementary Fig. 9g). Together, these findings suggest that increased hippocampal expression of C5aR1 and CR3 is associated with enhanced cognitive function following CR2-Crry treatment after rmCHI.

### Proteomic analysis after rmCHI revealed complement-dependent changes in proteins associated with wound healing, phagocytosis, and degenerative disorders

**Fig. 6:**
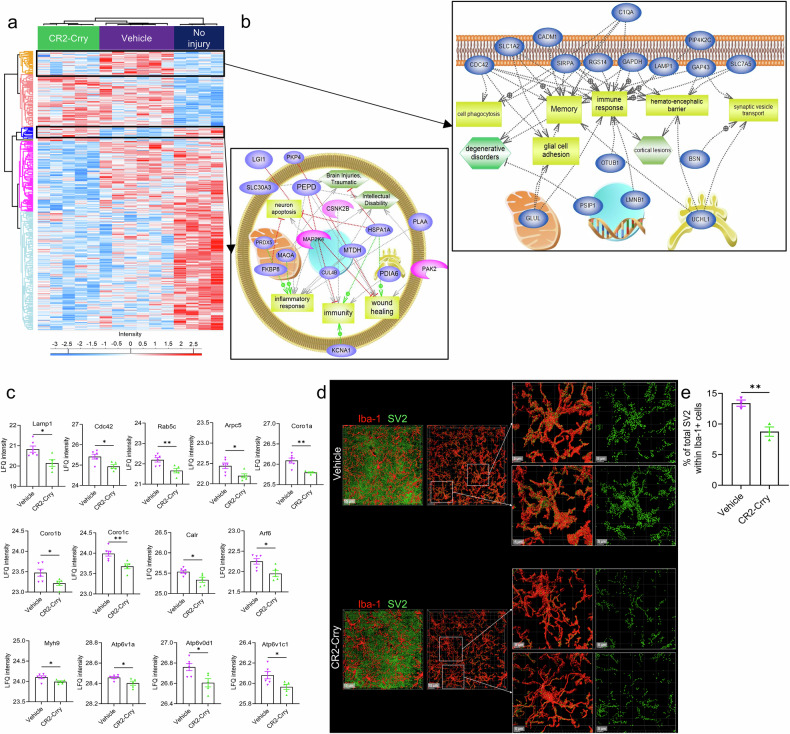
Proteomic analysis of rmCHI brains reveals complement-dependent changes in proteins associated with wound healing, phagocytosis, and degenerative disorders. **a** Heatmap of protein expression in the brains of non-injured mice (*n* = 4) and rmCHI mice treated with CR2-Crry (*n* = 5) or vehicle (*n* = 6) at 21 days after the last impact and based on their LFQ expression value. **b** Pathway analysis of two highlighted clusters from panel A. Only pathways unique to these clusters are shown. Also shown in the 6b is the protein sub-cellular localization along with the corresponding pathway(s) it is implicated in. **c** LFQ intensity of phagocytosis-related proteins in vehicle-treated (*n* = 6) or CR2-Crry-treated (*n* = 5) animals from the proteomics analysis. Statistical analysis was performed via an unpaired t-test. Mean +/- SEM. *= *p* < 0.05, **= *p* < 0.01. d) Representative 63x images of microglia (red) and SV2 synapses (green) from the CA3 region in mice euthanized at 21 days after the last impact for both vehicle and CR2-Crry conditions. In addition, several cells per condition are magnified to show the extent of SV2 internalization by Iba1+ cells. e) Quantification of the percentage of total SV2 synapses within Iba1+ cells in the CA3 region of the hippocampus in vehicle-treated and CR2-Crry-treated animals at 21 days after the last impact. The number of replicates was three animals per condition, and each value corresponds to the average of the %SV2 in the Iba1+ cells in the CA3 region from both hemispheres of the animal. Statistical analysis was performed via an unpaired t-test. Mean +/− SEM. **= p < 0.01

We performed label-free quantification (LFQ) proteomics on brain extracts from non-injured mice and from rmCHI mice treated with CR2-Crry or vehicle. The brains were extracted 21 days after the last (12th) impact. A total of 595 proteins with a *p*-value < 0.05 were z-scored and grouped by hierarchical clustering via Pearson correlation with k-means pre-processing. Hierarchical clustering yielded 6 different clusters of proteins with different expression patterns across the three cohorts. The clusters are color-coded on the Y-axis of the heatmap in Fig. 6a and are as follows: cluster 549 (grey), cluster 576 (blue), cluster 586 (sky blue), cluster 588 (pink), cluster 589 (orange), and cluster 590 (magenta). The full list of 595 proteins is available in Supplementary Dataset 1. To obtain a better understanding of the cellular processes in which the proteins of each cluster are involved, we performed subnetwork enrichment pathway analysis **(**Fig. 6b**)**. For each cluster, we obtained the top 100 significant pathways in which the proteins of that cluster are involved (Supplementary Dataset 2). To identify the unique pathways for each of the clusters, we performed a Venn analysis, as shown in Supplementary Fig. 10. A list of the unique and common pathways for the clusters is shown in Supplementary Dataset 3.

Next, we focused on clusters 589 and 576, which represented proteins upregulated (cluster 589) and downregulated (cluster 576) in the vehicle-treated group relative to CR2-Crry-treated and non-injured groups. We plotted several of the pathways unique to these clusters along with their corresponding proteins (Fig. 6b). Cluster 589 was associated with pathways such as immune response (Slc7a5, Glul, Lmnb1, Rgs14, Cdc42, Cadm1, Lamp1, Otub1, Sirpa, and C1qa), phagocytosis (Cdc42 and C1qa), and memory function (Slc1a2, Glul, Rgs14, Cdc42, Cadm1, C1qa, Sirpa, Uchl1, Gap43). Notably, the upregulation of C1QA (a classical pathway complement component upstream of C3 activation) in vehicle-treated animals and its downregulation in both CR2-Crry-treated and non-injured mice further support the involvement of complement in rmCHI pathology. To gain mechanistic insight into the effects of complement inhibition, we analyzed the proteomic dataset, identifying proteins whose expression levels were significantly altered between vehicle- and CR2-Crry–treated conditions. This analysis revealed several phagocytosis-related proteins that were significantly reduced in CR2-Crry–treated mice compared with vehicle-treated counterparts, including Lamp1,^35–37^ Cdc42,^38^ Rab5c,^39–41^ Arpc5,^42^ Coronin family proteins (Coro1a, Coro1b, Coro1c),^43–45^ Calr,^46,47^ Arf6,^48^ Myh9,^49^ and V-ATPase proteins (Atp6v1a, Atp60d1, Atp6v1c1).^50–53^ As shown in Fig. 6c, the expression of all above mentioned proteins was significantly decreased following CR2-Crry treatment.

To extend these findings, we performed immunofluorescence staining for synaptic markers and microglia in the hippocampus (specifically the CA3 region). We observed a reduced proportion of synapses engulfed by microglia in CR2-Crry-treated mice compared with vehicle-treated mice (Fig. 6d-e), supporting our hypothesis that cognitive impairment in vehicle-treated mice is driven, at least in part, by complement-mediated phagocytosis. Conversely, cluster 576 was downregulated in the brains of vehicle-treated mice but upregulated CR2-Crry-treated and non-injured mice. This cluster contained proteins associated with pathways involved in immunity (Kcna1, Hspa1a, Pdia6, Map2k4, Csnk2b, and Pepd), the inflammatory response and oxidative stress mitigation (Maoa, Plaa, Hspa1a, Map2k4, Prdx5, Pepd, Mtdh), and wound healing (Pkp4, Lgi1, Hspa1a, Mtdh, and Pepd).

**Fig. 7:**
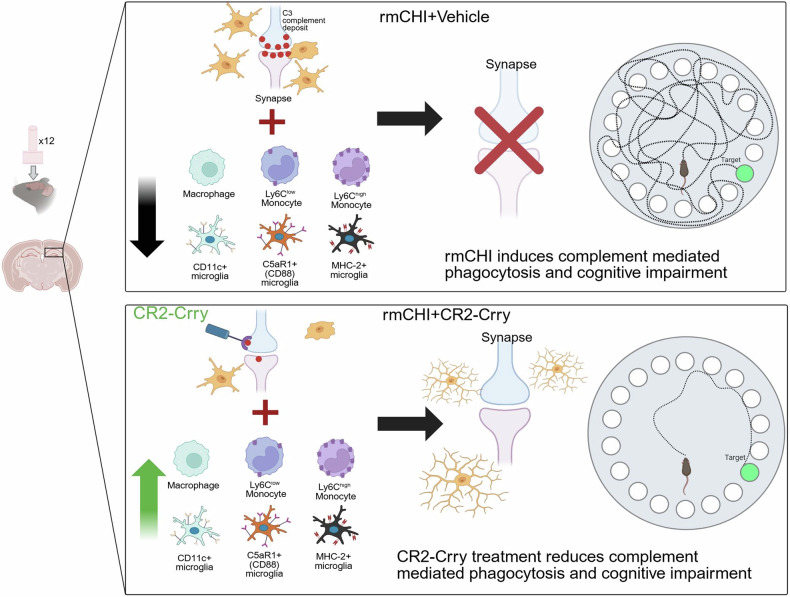
Schematic summarizing the role of complement in rmCHI. The schematic depicts the contribution of complement deposition and immune cell alteration to synapse preservation and cognitive impairment, all of which are halted upon complement inhibition via CR2-Crry. Created in BioRender.com

Taken together, these findings support our central hypothesis that complement activation following rmCHI drives aberrant phagocytic activity in brain regions critical for cognitive function and that site-targeted complement inhibition with CR2-Crry mitigates this pathological process. Alongside the immune landscape revealed by CyTOF analysis, our data suggest that the neuroimmune response induced by CR2-Crry treatment promotes the resolution of rmCHI-associated neuronal damage through the modulation of the complement system (Fig. 7).

## Discussion

A limited number of studies have examined the role of the complement system in CHI, primarily using single-hit paradigms. These studies consistently demonstrated that multiple complement components contribute to post-traumatic neuroinflammation. For example, in a weight drop model, mice lacking the alternative pathway presented decreased C5a serum levels with fewer TUNEL-positive (dying) neurons.^54^ The same group also demonstrated that an inhibitor of the alternative pathway (CR2-fH) reduced C3 deposition, decreased neuronal cell death, and suppressed microglial activation after a single-hit severe CHI,^13^ indicating an essential role for the alternative pathway in promoting secondary injury. Additionally, systemic C3 inhibition via Crry-Ig improved cognitive outcomes and decreased neuronal destruction in hippocampal sublayers after CHI.^55^ Similarly, soluble Crry expression by astrocytes reduced neurological impairment and improved blood-brain barrier (BBB) function in a single-impact CHI model.^11^ A role for the terminal complement activation product, the membrane attack complex (MAC), has also been implicated in post-CHI pathology since mice lacking the MAC inhibitor CD59 exhibited worsened neurological outcomes and increased brain tissue destruction.^12^ Additional studies investigating induced neural stem cells (iNSCs) in the context of CHI have shown that iNSC grafts reduce serum C3a and C5a, as well as brain tissue deposition of C3d and C9 following single-impact CHI.^56^ The same group also demonstrated that iNSCs treated with serum from mice subjected to CHI enhanced the expression of membrane Crry in iNSC-derived astrocytes, which was linked with a decrease in neuronal apoptosis.^57^ Additionally, C3d+ microglial clusters were detected in mice subjected to severe CHI.^58^ All of these preclinical studies examining complement in CHI have employed single-hit models that typically result in moderate to severe injury. However, the role of the complement system in repetitive mild CHI remains largely unexplored, despite its particular relevance to head injuries sustained in contact sports and military settings.

Many variations in mouse models of rmCHI have been described that differ in the type and location of impact, number of impacts, and interval between impacts (reviewed in ref. ^59^). Most preclinical models of CHI do not exceed 5 impacts, making them suboptimal for modeling repetitive head trauma sustained in contact sports, where athletes may sustain multiple impacts within a single game or training session.^59,60^ To better mimic these conditions, we evaluated outcomes following 4, 8, and 12 mild impacts, ultimately selecting the 12-hit paradigm to investigate the role of complement and the effects of complement inhibition. Impacts were administered at 48-hour intervals, as shorter intervals have been associated with more severe injury outcomes.^61^ During model optimization, we assessed both cognitive performance and immune cell dynamics. While neurobehavioral performance did not differ substantially between the 8- and 12-hit groups, the 12-hit paradigm elicited the most pronounced alterations in both immune and nonimmune cell populations. In particular, we observed greater numbers of Ly6C+monocytes and MerTK+ macrophages in the brains of mice subjected to 12 impacts compared with the 4- and 8-impact groups or non-injured controls. Notably, flow cytometry analyses confirmed that these changes were attributable to repeated head impacts rather than repeated exposure to anesthesia.

It is plausible that the monocytes and macrophages observed in the brains of both non-injured and 12-impact animals originate from the meninges and/or from the skull bone marrow. However, their functional states may differ significantly in response to the distinct inflammatory cues triggered by repetitive head injury. In this context, the level of injury in the 4- and 8-impact groups may be sufficient to reduce the population of “normal” mononuclear phagocytes circulating in the meninges but insufficient to induce a sustained inflammatory response comparable to that observed in the 12-impact group. Based on our analyses, we determined that a 12-hit rmCHI model was optimal for capturing measurable differences in both cognitive performance and neuroimmune alterations. Therefore, using this model, we employed three distinct multiomic approaches to characterize the role of complement in TBI pathogenesis, focusing on transcriptomic and proteomic brain profiles as well as the neuroimmune response.

Using mass cytometry, we identified at least five phenotypically distinct microglial subpopulations, and these findings align with recent studies demonstrating the functional and phenotypic heterogeneity of microglia in various contexts.^62^ Notably, complement inhibition following rmCHI resulted in increased frequencies of patrolling microglia, as well as MHC-II+ and CD11c+ microglia. Patrolling microglia exhibited a downregulation of core markers, such as CX3CR1 and P2Y12R, indicating a less inflammatory phenotype and that would be expected to be protective against secondary injury after TBI. For example, P2YR12 senses ATP released upon tissue damage, leading to decreased microglial ramification and inflammasome activation with increased IL-1b production.^63^ Therefore, the increased frequency of this patrolling microglial subpopulation in CR2-Crry-treated TBI animals likely reflects the expansion of a less inflammatory population that may help limit post-TBI neuroinflammation and secondary injury.

Although complement inhibition increased the frequency of MHC-II⁺ microglia, the functional significance of this subset in neuroinflammation remains unresolved. Their role in neuroinflammation has been studied primarily in the context of multiple sclerosis, for which MHC-II risk alleles have been identified.^64^ Interestingly, a recent study by Jung and collaborators^65^ revealed that in mouse models of multiple sclerosis (EAE), the ablation of MHC-II on microglia negatively impacts the remission phase. The authors concluded that MHC-II is critical for maintaining the regulatory identity of Tregs, and in its absence, the balance is shifted toward T cells that fuel the inflammatory process in EAE.^65^ A protective role for MHC-II-expressing microglia has also been described in stroke, where MHC-II constructs confer an anti-inflammatory state to microglia, ultimately resulting in reduced infarct volume and improvement in long-term neurological deficits in mouse models of ischemic stroke.^66^ In our study, the increase in MHC-II+ microglia observed in the CR2-Crry-treated animals was accompanied by elevated levels of effector memory CD4 + T cells and a significant reduction in CD8 + T cells compared with vehicle-treated animals. These findings are particularly noteworthy, as previous studies have shown that antigen-experienced memory effector T cells are constitutively present in the brain throughout adult life in naïve mice, where they play key roles in immune surveillance and in limiting microglial inflammatory potential.^34^ Although CD4 + T cells are generally less abundant than CD8+ T cells in the brain parenchyma, they exhibit a markedly reduced capacity for stimulus-driven cytokine production, suggesting a more regulatory or surveillance-oriented function. In contrast, the increased proportion of naïve CD8+ T cells found in vehicle-treated mice may reflect injury-induced recruitment of antigen-inexperienced CD8+ T cells, which could later transition to cytotoxic cells and exacerbate inflammation. This phenomenon has been previously described in a mouse model of CCI, where cytotoxic CD8+ T cells were shown to inhibit the development of anti-inflammatory CD4+ T cell responses and contribute to chronic neuroinflammation and cognitive dysfunction up to 32 weeks post TBI. Accordingly, CD8+ T cell depletion, but not CD4+ T cell depletion, prevented TBI-associated neurologic dysfunctions.^67^ Taken together, our findings suggest that in rmCHI, cross-talk between MHC-II+ microglia and CD4+ T cells may be important for dampening proinflammatory immune responses and limiting the accumulation of antigen-inexperienced CD8 + T cells with cytotoxic potential.

Consistent with our previous findings in a contusive CCI model of severe TBI, we identified CD11c+ (complement receptor 4 [CR4]) microglia in the brains of mice subjected to rmCHI.^30^ Notably, treatment with CR2-Crry increased the frequency of CD11c+ microglia, suggesting a role for this subset in the subacute phase of injury. This finding aligns with reports of their emergence in other neuroinflammatory conditions, such as experimental autoimmune encephalomyelitis, stroke and AD.^68–71^ Interestingly, recent studies on the ontogeny of mononuclear phagocytes in neurodegeneration have suggested that CD11c+ DAM in AD mice undergo transcriptional reprogramming to resemble neonatal and early postnatal CD11c+ microglia.^72^ Particularly, a distinct population of osteopontin-expressing CD11c⁺ microglia has been shown to play a neuroprotective role by clearing amyloid-β plaques without inducing the production of proinflammatory cytokines.^73^ In support of their relevance in human pathology, CD11c+ microglia have been observed to cluster around neuritic plaques in the hippocampus and frontal cortex of individuals with intermediate to high AD pathology.^74^ In addition to their role in AD, CD11c+ microglia have been shown to have protective functions in models of neuropathic pain and stroke,^75^ further supporting their context-dependent, beneficial role in various CNS disorders.

An intriguing observation following complement inhibition was the significant upregulation of C5aR1-expressing microglia, which correlated with improved performance on the Barnes maze task. In contrast, in the context of neurodegenerative diseases such as AD, C5aR1 expression has been associated with neurotoxic microglial clusters. Notably, pharmacological C5aR1 antagonism via PMX205, which is ineffective at reducing the amyloid plaque burden, was able to prevent cognitive decline.^76^ In support of our results in the rmCHI model, we previously reported that C5aR1 antagonism in a CCI model reduced peripheral immune cell infiltration in the injured brain but failed to improve cognitive performance.^30^ These apparent discrepancies may reflect fundamental differences in the mechanisms and types of inflammation that underlie acute traumatic brain injury versus chronic neurodegenerative disease.

Treatment with CR2-Crry after rmCHI also increased the frequencies of Ly6C^high^ and Ly6C^low^ monocytes, as well as conventional dendritic cells (cDC). Infiltrating monocytes are known to contribute to tissue repair and secrete chemokines that recruit astrocytes during the recovery phase.^77^ Although recent reports have shown alterations in cDC differentiation and distribution in response to TBI, their role in the injured brain remains poorly understood.^78^

Our finding that treatment with CR2-Crry results in an increase in several immune cell populations is intriguing. However, this accumulation of certain cell types should not be interpreted as a direct indicator of heightened inflammation. Although an inflammatory response can contribute to neuronal death and neurodegeneration, it is also essential for tissue repair, including the removal of cell debris and the activation of repair programs. In support of this dual role, recent work has demonstrated that during cerebrovascular injury, whether induced by stroke, neurodegeneration, TBI, or autoimmune disease, infiltrating monocytes are the primary source of IL-6 and are required for instructing microglia to repair the damaged vasculature and promote recovery.^79^ Furthermore, Frieler and collaborators reported that depletion of monocytes/macrophages during TBI results in a marked increase in proinflammatory gene expression in the injured brain.^80^

Overall, this duality of a post-TBI immune response may explain why attempts to target immune activation in TBI patients have often proven ineffective and, in some cases, even harmful.^81–83^ Notably, the neuroimmune landscape following repetitive concussive TBI has not been well characterized. Indeed, most preclinical studies have focused on models of moderate to severe TBI, often employing controlled cortical impact paradigms. These models are characterized by robust neuroinflammation and immune cell recruitment, especially in the acute phase, and they model injuries distinct from repetitive, mild closed head trauma.^15,30,67,84^ Compared with severe and contusive forms of injury, repetitive concussive TBI results in less overt structural damage and a milder inflammatory response.

In light of our findings, we propose that the increase in immune cells in CR2-Crry animals is not deleterious but instead plays a role in limiting damage and promoting tissue repair. It is possible that, in addition to increasing immune cell numbers, CR2-Crry treatment may reprogram these cells toward protective and reparative functions. Future studies using cell type-specific deletions will help better define the roles of microglia and other myeloid cell populations in post-TBI pathology and outcomes.

In addition to modulating immune responses, complement inhibition following rmCHI resulted in major alterations in proteomic and transcriptomic profiles. Neuroproteomics and systems biology have previously been applied to murine models of CNS injury, including TBI^85^ and stroke.^86,87^ Using shotgun proteomics, we identified multiple protein clusters that were differentially expressed among non-injured, vehicle-treated, and CR2-Crry–treated groups. One such cluster, which is overexpressed in vehicle-treated animals compared to non-injured and CR2-Crry-treated animals, contains Ubiquitin C-terminal hydrolase-L1 (UCHL1), a brain-derived biomarker that has been detected in cerebrospinal fluid (CSF) and is associated with TBI.^88^ Given the translational relevance of this finding, the FDA recently approved the combined use of UCHL1 and GFAP as biomarkers for assessing intracranial injury in patients with mild TBI.^89^ Efforts to identify postmortem TBI biomarkers in the serum and urine of patients have expanded this field. Olczak et al. reported elevated levels of progranulin (PGRN), GFAP, and MAPT in both the serum and urine of postmortem TBI patients compared with sudden death controls.^90,91^ In our dataset, C1qa, a component of the classical complement pathway, was part of the protein cluster upregulated in vehicle-treated mice. This finding aligns with clinical findings showing that increased serum C1q levels in TBI patients correlate with injury severity^92^ and that C1q is upregulated in the saliva of pediatric brain injury patients.^93^ C1q has also been implicated in cognitive dysfunction across multiple neurological conditions, including AD, cranial radiation therapy, and epilepsy. In a mouse model of AD, C1q deletion resulted in reduced glial activation and attenuated synaptic loss at 16 months of age, demonstrating a deleterious role of C1q in disease progression.^94^ Similarly, in a mouse model of cranial radiation therapy, microglia-specific C1q deletion protected against cognitive deficits compared with irradiated wild-type controls.^95^ Following TBI, C1q is upregulated in the corticothalamic system, where it contributes to neuronal loss and chronic inflammation and is associated with the emergence of epileptiform activity and the disruption of sleep spindles.^17^ Collectively, these studies suggest a conserved role for C1q in mediating neuroinflammatory responses across diverse neurological diseases and injury states, including TBI.

A focused analysis of the proteomic dataset revealed a set of 13 phagocytosis-related proteins, including Lamp-1, that were significantly reduced in CR2-Crry-treated animals compared with vehicle-treated animals. These findings, together with the observed reduction in SV2 internalization in CR2-Crry-treated animals, support our hypothesis that rmCHI induces complement-dependent synapse elimination, which can be prevented by treatment with CR2-Crry. At the transcriptomic level, iPathway analysis highlighted that among the top 20 Gene Ontology (GO) biological processes enriched in DEGs in the brains of CR2-Crry-treated vs. vehicle-treated rmCHI mice, several were linked to neurogenesis, synapse organization, dendritic spine development, neurotransmitter transport, and neuronal development. Taken together, our multiomic analyses define a mechanistic role for the complement system in driving neuroinflammation after repetitive mild CHI and demonstrate that complement inhibition is associated with the activation of reparative programs that may prevent progressive cognitive decline. These findings carry important therapeutic implications for the treatment of repetitive head trauma.

Complement inhibitors have gained traction as promising therapeutic candidates for TBI and other neurological disorders. Several inhibitors targeting C1, C3, C5, and C5a have undergone development and received approval for clinical applications, although only anti-C5 therapies have received approval for CNS indications (generalized myasthenia gravis and neuromyelitis optica spectrum disorder). The landscape of complement-targeted therapeutics is rapidly expanding, with about 16 complement drugs currently approved for human use numerous others undergoing clinical evaluation.^96,97^ Notably, most existing drugs systemically inhibit complement, which can carry risks of broad immunosuppression. In contrast, site-targeted inhibitors, such as the agent used in our study, offer the advantage of delivering the complement inhibitor specifically to sites of complement activation, thereby maximizing local bioavailability while minimizing systemic effects.^98^ A site-targeted complement inhibitor in clinical development is ADX-097 (Q32Bio), a humanized targeted C3 inhibitor that binds the same ligand as CR2-Crry, the murine inhibitor used in our study.^99^ ADX-097 has shown good safety and tolerability in Phase 1 trials and is currently ready for Phase 2 clinical evaluation. Together, these developments underscore the translational potential of our findings and support the rationale for complement-based therapies in TBI.

Despite the strengths and novel insights provided by our study, we acknowledge several limitations. First, our 21-day endpoint reflects a subacute time point. Given the chronic progression of TBI-related neurodegeneration, longer-term studies at 6, 12, and 18 months post-injury are essential to fully assess the long-term consequences of rmCHI, its potential link to neurodegenerative disorders such as AD, and the sustained efficacy of complement inhibition. Preliminary data from our laboratory indicate that cognitive deficits persist for at least 6 months post-rmCHI and are preventable by CR2-Crry treatment, and ongoing studies are evaluating 1- and 2-year outcomes. Second, our current study was performed exclusively in male mice. While the incidence of TBI is greater in males than in females,^100^ increasing evidence supports a significant influence of sex on immune responses and disease progression. It is therefore imperative to assess sex as a biological variable in future studies of rmCHI and complement inhibition. Finally, while CR2-Crry effectively inhibits complement activation, it blocks all three activation pathways (classical, lectin, and alternative), making it difficult to delineate which specific pathway mediates injury in the rmCHI model. In a prior study using controlled cortical impact (CCI), we demonstrated that the alternative pathway was the primary driver of complement activation by comparing pathway-specific inhibitors: CR2-fH (alternative pathway), CR2-CD59 (terminal pathway), and CR2-Crry (all pathways).^14^ Similar pathway-specific investigations in the rmCHI model will be necessary to refine and optimize therapeutic strategies.

In conclusion, the studies presented here are the first to establish a role for the complement system in driving the neuroimmune response and secondary injury following rmCHI. Through multiomic analyses, we demonstrated that rmCHI induces a robust immune response, alters microglial subpopulations, and activates transcriptomic and proteomic pathways associated with neurodegeneration, including increased complement-mediated synaptic phagocytosis. Notably, changes associated with rmCHI, including cognitive impairment, were either fully or partially reversed by complement inhibition administered after the injury cycle. Collectively, these results highlight the therapeutic potential of complement-targeted strategies for mitigating the long-term pathological consequences of repetitive mild traumatic brain injury.

## Materials and methods

### Animal care and housing

Adult male C57BL/6 mice (10 weeks old) were purchased from the Jackson Laboratory. After one week of acclimation, the animals were subjected to different paradigms of injury as described below. All the animal experiments were performed after approval by the Institutional Animal Care and Use Committee (IACUC) at the Medical University of South Carolina and Ralph H. Johnson VA Medical Center. The animals were housed in standard-sized cages with a 12:12 h light: dark cycle, and experiments were performed during the light cycle.

### Study Design

This study has two main parts: (1) rmCHI model optimization and characterization, and (2) investigation of the role of complement in an optimized rmCHI model. For model optimization, three injury paradigms consisting of 4, 8, or 12 total impacts were investigated (refer to Fig. 1). The time between impacts was 48 hours. The animals were sacrificed 14 days after the final impact, and the brains were extracted for flow cytometric or immunohistological analysis as described below. To study the role of complement in rmCHI, the 12-hit paradigm was used, which was determined to be optimal. For this part of the study, the animals were euthanized 21 days after the final impact, and the brains were extracted for CyTOF, RNA-seq, proteomic, and immunohistological analyses as described below.

### Closed head injury (CHI) procedure

The animals were anesthetized via a ketamine (75 mg/kg)/xylazine (7.5 mg/kg) cocktail, after which the head of each animal was shaved and placed in a sponge head mold. The head mold was custom-built using a moldable glue (Sugru) that fit the head of the C57BL/6 mice used in our study and prevented lateral movements during impact. The impactor tip was custom-made using silicon, and the 9 mm diameter ensured that the center of the impact corresponds to the midway between the interfrontal and lambdoid sutures. Injuries were performed via a pneumatic impactor device (Infinite Horizon, Precision Scientific, Inc.) equipped with a custom-made rubber tip. The impact was delivered to the center of the head and midway between the ears. The impactor parameters were as follows: tip width, 0.9 cm; depth, 4 mm; velocity, 4 m/s; and dwell time, 200 ms. A schematic of the estimated location and extent of injury directly beneath the impactor tip is shown in Fig. 1a. Non-injured controls received the same manipulations as the injured animals except for the mechanical impact. At the experimental endpoints, animals that had skull fractures were excluded from the study. Before impact injury, the mice were randomly assigned to treatment groups that were coded for each animal. The codes were not accessible to the experimenter performing the CHI or to the individuals who performed the behavioral tasks. We also investigated the effect of repeated anesthesia alone (i.e., no injury) via the same schedule as in the 12-impact paradigm and determined that anesthetic exposure alone did not contribute to the differences we observed in injured animals.

### Behavioral testing

NSS: Short-term motor and sensory deficits were measured via a 10-item neurobehavioral assessment scale (the mNSS-R) as previously described.^101^ For NSS, n = 8–13 mice/group were used (more details in the figure legends). Barnes Maze: This task was used to assess cognitive performance, specifically spatial learning and memory retention, as previously described.^14,16,102^ This task commenced on day 7 after the final hit for all experimental groups in the manuscript. Briefly, the mice were placed on a circular platform containing a target escape hole and allowed to explore for 5 min per trial. The spatial learning phase consisted of 5 consecutive days of training with 2 trials per day. Once completed, the animals were given a 2-day break, and then, memory retention was assessed. All trials were video recorded, and the data were analyzed via the Noldus EthoVision XT system. For the Barnes Maze, n = 8–11 mice/group were used (more details in the figure legends). Y-maze: This task was used to assess spatial working memory and was performed as previously described.^103^ Briefly, the mice were placed into one arm of a Y-maze for one 5 min trial. Using ANY-maze behavior tracking software (Stoelting), correct alternations per mouse during the trial were recorded. An alternation was deemed correct when the mouse entered a series of 3 different arms without re-entering a previously explored arm. For the Y-maze test, *n* = 13–15 mice/group were used (more details in the figure legends).

### Brain procurement and processing

At either 24 h, 14 days, or 21 days after the final impact, the mice were subjected to isoflurane overdose followed by intracardiac perfusion with ice-cold PBS. For immunohistology studies, perfusion with ice-cold PBS was followed by 4% paraformaldehyde dissolved in PBS, and the brains were stored at 4 °C in 30% sucrose as previously performed.^16^

### Immunofluorescence and immunohistochemistry

Microglia, CR3, C5aR1, synapses (SV2), and macrophages (F4/80) were stained by immunofluorescence via the free-floating method with 40 µm sections as previously described.^16^ The primary antibodies used were as follows: Iba-1 (Invitrogen, Cat.#: PA5-18039, 1:200), F4/80 (BioLegend, Cat.#: 123101, 1:150), CD88 (C5aR1) (Bio-Rad, Cat.#: MCA2456, 1:100), cd11b (CR3) (Abcam, Cat.#: ab133357, 1:100), and SV2 (Developmental Studies Hybridoma Bank, AB_2315387, 1:100). The secondary antibodies used were as follows: donkey anti-goat IgG Alexa Fluor 647 (Invitrogen, Cat. #: A32849, 1:200), donkey anti-rat IgG Alexa Fluor 555 (Invitrogen, Cat. #: A48270, 1:200), donkey anti-rabbit IgG Alexa Fluor 555 (Invitrogen, Cat. #: A31572, 1:200), and donkey anti-mouse IgG Alexa Fluor 488 (Invitrogen, Cat. #: A21202, 1:200). For microglial morphology analysis, confocal imaging was performed via a Leica SP5 microscope at 63x magnification with a 1024 × 1024 frame size. Images were acquired via the Z-stack feature and set at 1 µm per slice to cover the 40 µm thickness of the tissue. Images were then processed on Labkit AI for background removal and analyzed via the 3D Morph MATLAB-based script.^104^ The threshold of all the images was set to 0.2, and the noise filter was set to 300. The cells touching the x and y borders were not included in the analysis. Individual microglia were analyzed for the ramification index, cell territory volume (µm^3^), and cell volume (µm^3^). For synapse internalization analysis, the same imaging parameters and Leica SP5 confocal microscope described above were used. A 30 µm region was analyzed per image via Imaris 10.2 (Oxford Instruments). To quantify the internalization of SV2+ synaptic puncta within Iba1+ microglia, an Iba1 surface was first generated from the Iba1 channel. SV2 spots were then identified via spot features and masked to the Iba1 surface to isolate only those SV2 spots located within Iba1+ cells.

For intensity analysis of microglia, macrophages, CR3, and C5aR1 in the hippocampal regions, images were acquired at 20x magnification via a Keyence BZ-X710 microscope. For F4/80 staining, 4 z-stack images per animal were captured with a step size of 1 µm per slice. For CR3 and C5aR1, a tile scan at 20x magnification of the full hippocampus was acquired and stitched. After acquisition, the images were quantified via NIH ImageJ. Colocalization analysis was conducted via the DiAna plugin.^105^

C3 deposition was detected by IHC using a free-floating method with 40 µm sections. Briefly, the brain slices were washed 3 times with TBS on a shaker, followed by incubation in TBS containing 1% hydrogen peroxide for 30 min. The sections were then washed again with TBS, followed by the addition of Dako washing solution. The sections were blocked in 2.5% goat serum for 1 hour at room temperature and then incubated overnight at 4 °C with an anti-C3 Ab (Abcam, cat. #: ab11862, 1:500). The following day, the sections were washed 3 times with Dako washing solution, followed by incubation with the secondary antibody for 1 h at room temperature (HRP goat anti-rat vector labs, cat # MP-7404-50). Following washing, the sections were incubated with DAB substrate (Vector Labs cat # SK-4105), and the reaction was terminated with H2O before mounting. For analysis, the sections were imaged at 40X via a Keyence BZ-X710 microscope via the Z-stack feature and set at 1 µm per slice to cover the 40 µm thickness of the tissue. Four images per brain slice were acquired, and the signal intensity was analyzed via ImageJ (NIH) via the integrated density feature. For all strains in the manuscript, n = 3 mice/group were used.

### Statistical analysis

Statistical analysis was carried out using GraphPad Prism (version 10). All data were analyzed for normal distribution before performing the analyses. The unpaired t-test was used for comparisons between two groups. Comparisons of multiple groups were carried out via one-way or two-way ANOVA (for multiple time points) followed by multiple comparisons analysis (using Bonferroni correction), and repeated measures ANOVA for matched data. Pearson’s correlation coefficient was used to compute correlations between the Barnes Maze and immunohistology. *P*-value and R^2^ were used to evaluate each correlation and are reported in the figures. All the statistical tests were two-sided, and P < 0.05 was considered statistically significant. Outliers were removed via the ROUT method on GraphPad Prism.

Other materials and methods are available in the supplementary information file.

## Supplementary information

Supplementary Material

Dataset 1

Dataset 2

Dataset 3

Supp. Video 1: No injury (all)

Supp. Video 2: No injury (single)

Supp. Video 3: 12hits (all)

Supp. Video 4: 12hits (single)

## Data Availability

The proteomics data have been deposited in the PRoteomics IDEntifications Database (PRIDE) and assigned the following accession number: PXD053772. Raw RNASeq data have been deposited on the NCBI database BioProject database with the submission ID SUB15667826 and BioProject ID PRJNA1335116.
