# Supplementary material for: Targeted complement inhibition ameliorates the pathological and cognitive outcomes in repetitive mild closed head injury
Source: Signal Transduct Target Ther. 2025 Nov 20;10:378. doi: 10.1038/s41392-025-02466-7 (PMC12630812; doi:10.1038/s41392-025-02466-7)
Supplement: Supplementary file 1 — Supplementary Material [file 41392_2025_2466_MOESM1_ESM.docx]

Supplementary Materials for

Targeted complement inhibition ameliorates the pathological and cognitive outcomes in repetitive mild closed head injury

Khalil Mallah, Carsten Krieg, Devin Hatchell, Nahla Hamouda, Tylar Roof, Stephen Walterhouse, Amer Toutonji, Davis Borucki, Christine Couch, Gary Hardiman, Firas Kobeissy, Silvia Guglietta*, and Stephen Tomlinson*.

Correspondence to: Dr. Stephen Tomlinson (tomlinss@musc.edu) and Dr. Silvia Guglietta (gugliett@musc.edu).

**This PDF file includes:**

Materials and Methods

Supplementary Figures 1 to 10

Supplementary Table 1

Captions for Supplementary Datasets 1 to 3

Captions for Supplementary Movies 1 to 4

**Other Supplementary Materials for this manuscript include the following:**

Supplementary Datasets 1 to 3

Supplementary Movies 1 to 4

**Materials and Methods:**

*Recombinant protein production and purification:* The complement inhibitor used in this study was CR2-Crry: complement receptor 2 fused with complement receptor 1–related gene/protein y. CR2-Crry was prepared as previously described and was confirmed to be endotoxin free^1^. Complement inhibitory activity was confirmed by zymosan assay^1, 2^. CR2-Crry was administered i.p. at 10 mg/kg.

*Single-cell preparation:* Single-cell suspensions were prepared from the brains of injured and non-injured mice as described previously but with minor modifications^3, 4^. Briefly, after euthanasia and transcardial perfusion with 15 ml of cold PBS, the brains and meninges were gently minced via a scalpel and digested in Hibernate-A medium supplemented with 0.4 mg/ml collagenase IV (Sigma‒Aldrich) for 30 minutes at 37°C on a magnetic stirrer. The cell suspensions were then filtered, and subjected to a Percoll gradient centrifugation for 30 minutes at 4°C to remove myelin and debris. Finally, the cells were washed in cold PBS, counted, and used for flow cytometric or mass cytometric analysis. For the flow experiments, n=8–11 mice/group were used. For the mass cytometry experiments, a minimum of 5 mice/group were used.

*Flow cytometry and mass cytometry:*

For flow cytometry analysis, single-cell suspensions (see above) were stained with a cocktail of antibodies against CD45.2 (clone 104), CD11b (clone M1/70), CD3 (clone 145-2C11), Ly6G (clone 1A8), Ly6C (clone AL21), F4/80 (clone T45-2342), and MerTK (clone BB14-16), together with a live/dead fixable marker (Molecular Probes). This cocktail of antibodies was used to identify and differentiate between the following cellular populations: immune cells (T cells, monocytes, neutrophils, and macrophages), resident mononuclear phagocytes (microglia and border-associated macrophages), and nonimmune cells. All the antibodies were purchased from BD Biosciences unless otherwise specified. After staining, the cells were washed in FACS buffer (5% FBS in PBS and 2.5% EDTA), fixed in 1% PFA, and data were acquired on a BD Fortessa X-20 analytic flow cytometer. Analysis was performed via FlowJo V10 analysis software.

For mass cytometry, single-cell suspensions were analyzed via antibodies that were labeled in-house via either antibody-labeling kits and protocols according to the manufacturer’s instructions (Standard BioTools) or antibodies purchased from Standard BioTools. Prior to labeling and using for mass cytometry, antibodies were individually titrated and optimized for concentration by flow cytometry. We followed the protocol of Mei *et al*. and used six palladium metal isotopes for live-cell barcoding of samples with CD45.2^5^. The barcodes used in this study consisted of unique combinations of 3 out of 6 antibody‒heavy metal complexes, yielding 20 possible barcodes. Briefly, 1 × 10^6^ cells from individual samples were incubated with the respective CD45-Pd antibodies in cell staining buffer at 4°C, washed, and combined into one sample. To identify dead cells, 2.5 μM cisplatin in PBS was added for 2 min at room temperature. The composite sample was stained with a cocktail of 34 antibodies (**Supplementary Table 1**), washed and incubated with DNA-intercalating solution (Iridium; Sigma) in MaxPar Fix/Perm buffer (Standard BioTools) overnight at 4°C. Before data acquisition, the samples were washed twice with Milli-Q water. Barcoded composite samples were acquired on a Helios mass cytometer (Standard BioTools). The quality control and tuning processes for the Helios were performed daily before acquisition. Data from different days and across acquisition times were normalized by adding five-element beads to the sample immediately before acquisition and using MATLAB-based normalization software, as described previously^6, 7^. The mass cytometry data were analyzed following a previously published workflow ^8^. Data were acquired from ~ 62,000 cells from each sample.

*RNA extraction from brain tissue:* Mice were euthanized by isoflurane overdose and cervical dislocation, followed by transcardiac perfusion with ice-cold PBS. The brains were harvested and immediately stored in RNAlater solution at 4°C for two days prior to being transferred to -20°C until further processing. For RNA extraction, whole brains were first mechanically homogenized in TRIzol via a tissue homogenizer. Chloroform was added at a 1:5 ratio to the homogenized tissues, and after centrifugation, the supernatant was loaded onto Qiagen Mini Kit columns and processed according to the manufacturer’s instructions (RNeasy Tissue Mini Kit, Qiagen). We evaluated the quality and integrity of the RNA via a bioanalyzer (Agilent Technologies), and we only used RNA with an integrity number (RIN) >8 for bulk RNA-seq analysis from n=3 mice/group.

*Bulk RNA-seq:* Messenger RNA was purified from total RNA via poly-T oligo-attached magnetic beads, and libraries were generated via the TruSeq RNA Kit (Illumina). The quality and concentration of the obtained libraries were evaluated via a bioanalyzer (Agilent Technologies) and a Qubit assay, respectively. The quantified libraries were pooled and sequenced on Illumina platforms at Novogene according to the effective library concentration and data amount. Clustering of index-coded samples was performed according to the manufacturer’s instructions. After cluster generation, library preparations were sequenced on an Illumina platform, and paired-end reads were generated.

Raw data (raw reads) in fastq format were first processed through in-house Perl scripts. In this step, clean data (clean reads) were obtained by removing reads containing adapters, reads containing poly-N and low-quality reads from the raw data. Moreover, the Q20, Q30 and GC contents of the clean data were calculated. All the downstream analyses were based on high-quality, clean data. The fragments per kilobase of transcript sequence per million (FPKM) base pairs sequenced was used to estimate gene expression levels, which takes into consideration the effects of both sequencing depth and gene length on the number of fragments, as previously described ^9^. Prior to differential gene expression analysis, for each sequenced library, the read counts were adjusted by the edgeR program package through one scaling normalization factor. Differential expression analysis was performed via the DESeq2 R package (1.20.0). The resulting P values were adjusted via Benjamini and Hochberg’s approach for controlling the false discovery rate. Genes with an adjusted P-value <=0.05 according to DESeq2 were considered differentially expressed. GO analysis was conducted to identify DEG at the biologically functional level. The identified DEGs were uploaded to the online software Advaita Bioinformatics to integrate functional genomic annotations. The GO biological processes were analyzed via the iPathwayGuide from Advaita Bioinformatics ^10^. A false discovery rate (FDR) cutoff of 5% and a minimum fold change of 1.5 (log2FC=0.6) were applied to determine differential expression.

*Label-Free Proteomics using LC‒MS/MS:* Whole-brain tissue was homogenized on ice in 9 M urea and 50 mM Tris (pH 8) supplemented with 100 units/mL Pierce Universal Nuclease. Debris was removed by centrifugation. Cysteines were reduced in 1 mM dithiothreitol (Thermo Scientific, Rockford, IL) at 55°C and alkylated in 5.5 mM iodoacetamide (Thermo Scientific) at room temperature for 30 minutes in the dark. The urea concentration was diluted with 50 mM ammonium bicarbonate to final concentration of 1.6 M, and the protein was sequentially digested with LysC for 3 h at room temperature and trypsin (Sigma, 100 ng) overnight at 37°C. The digestion was quenched with formic acid and the peptides were desalted via C18 Stage Tips. The eluted peptides were dried under vacuum and stored at -80°C. Peptides were analyzed by LC-MS/MS on an EASY nLC 1200 System (Thermo Scientific) coupled to an Orbitrap Fusion Lumos Mass Spectrometer (Thermo Scientific) with instrument control software v. 4.2.28.14. Peptides (2 µg) separated using a C18 reversed-phase column (Acclaim PepMap RSLC, 75 µm × 50 cm (2 µm, 100 Å) Thermo Fisher cat. # 164536) with a gradient of 5‒40% B for 180 min (Solvent A: 5% acetonitrile, 0.2% formic acid; Solvent B: 80% acetonitrile, 0.2% formic acid) at 300 nL/min. Data were acquired in DDA mode with a high-resolution FTMS survey scan (60,000) and mass range of m/z 375-1575 with a cycle time of 3 s. The automatic gain control target value was 4x10^5^ for the survey scan. HCD fragmentation was performed with a collision energy of 35%, isolation window of 1.6 m/z, and maximum injection time of 50 ms. Tandem mass spectra were acquired at 15,000 resolution. The monoisotopic precursor selection parameter was “peptide”. Dynamic exclusion was used for precursors with mass tolerance of 10 ppm for 25 sec. Advanced peak determination was enabled. Precursors with charge states that were undetermined, 1, or >7 were excluded.

Raw data acquired in 19 LC‒MS/MS analyses were searched against a murine UniProt protein database (17,082 sequences, updated October 2021) and including common contaminants via MaxQuant v1.6.14.0 (Max Planck Institute). Variable methionine oxidation, protein N-terminal acetylation, and fixed carboxyamidomethylation of cysteine were included. Two missed cleavages were permitted. A decoy database strategy was used to control for false positive with a 1% FDR threshold at the peptide spectral match and protein levels. The final mass tolerance was less than 4.5 ppm. A minimum of two peptides were required for protein identification including one unique peptide. Proteins were quantified and normalized by the MaxQuant LFQ algorithm with matching between runs enabled with a 0.7 min window to minimize missing values^11^.

Protein groups were filtered to remove potential contaminants and reversed database hits. After log2 transformation, the LFQ protein intensities were median normalized, and an arbitrary factor of 22 was added to avoid negative intensity values. Proteins were filtered to retain those quantified in 70% of the biological samples (no injury, n=4; vehicle, n=6; treated, n=5). Missing values were imputed using random values from a normal distribution downshifted by 1.8 with a width of 0.3 in Perseus v1.6.14.0 (Max Planck Institute). ANOVA was performed with a pval<0.05 threshold. Proteins (595) with a pval<0.05 were z scored and grouped by hierarchical clustering via a Pearson correlation with k-means preprocessing. A heatmap was generated to identify clusters of proteins coregulated under different treatment conditions. A Fisher exact test was performed to test for enrichment of GO annotations and Reactome Pathways in each cluster compared with all of the quantified proteins, with a Benjamini-Hochberg corrected p value of 0.02, as described previously ^12^.

*Subnetwork enrichment analysis:* Relationships between proteins were plotted via Elsevier’s Pathway Studio (version 11.0) and depicted via the Ariadne ResNet database^13^ as previously described^14^. Subnetwork Enrichment Analysis (SNEA) algorithm was employed to depict significantly altered biological pathways, which include the identified proteins; SNEA uses Fisher’s statistical test to detect any nonrandom associations between two categorical variables organized by a specific relationship. In this approach, a central “seed” is created from all relevant identities in the database, and then connections are built with associated entities on the basis of their relationship with the seed. For each protein, the GenBank ID was used to form experimental groups on the basis of the different conditions present for the analysis (i.e., each of the six clusters that resulted from the heatmaps). For each cluster, the top 100 statistically significant pathways were acquired, and a Venn analysis via InteractiVenn^15^ was conducted for all 6 clusters to obtain pathways that were unique to each cluster. The pathway networks were reconstructed and drawn on the basis of biological processes and molecular functions for each single protein, along with its associated targets within the pathway^16^.

References

1. Atkinson C*, et al.* Targeted complement inhibition by C3d recognition ameliorates tissue injury without apparent increase in susceptibility to infection. *J Clin Invest* **115**, 2444-2453 (2005).

2. Huang Y, Qiao F, Atkinson C, Holers VM, Tomlinson S. A novel targeted inhibitor of the alternative pathway of complement and its therapeutic application in ischemia/reperfusion injury. *J Immunol* **181**, 8068-8076 (2008).

3. Mrdjen D*, et al.* High-Dimensional Single-Cell Mapping of Central Nervous System Immune Cells Reveals Distinct Myeloid Subsets in Health, Aging, and Disease. *Immunity* **48**, 380-395 e386 (2018).

4. Mundt S, Mrdjen D, Utz SG, Greter M, Schreiner B, Becher B. Conventional DCs sample and present myelin antigens in the healthy CNS and allow parenchymal T cell entry to initiate neuroinflammation. *Sci Immunol* **4**, (2019).

5. Mei HE, Leipold MD, Schulz AR, Chester C, Maecker HT. Barcoding of live human peripheral blood mononuclear cells for multiplexed mass cytometry. *J Immunol* **194**, 2022-2031 (2015).

6. Krieg C*, et al.* Author Correction: High-dimensional single-cell analysis predicts response to anti-PD-1 immunotherapy. *Nat Med* **24**, 1773-1775 (2018).

7. Nowicka M*, et al.* CyTOF workflow: differential discovery in high-throughput high-dimensional cytometry datasets. *F1000Res* **6**, 748 (2017).

8. Toutonji A, Krieg C, Borucki DM, Mandava M, Guglietta S, Tomlinson S. Mass cytometric analysis of the immune cell landscape after traumatic brain injury elucidates the role of complement and complement receptors in neurologic outcomes. *Acta Neuropathol Commun* **11**, 92 (2023).

9. Mortazavi A, Williams BA, McCue K, Schaeffer L, Wold B. Mapping and quantifying mammalian transcriptomes by RNA-Seq. *Nat Methods* **5**, 621-628 (2008).

10. Ahsan S, Draghici S. Identifying Significantly Impacted Pathways and Putative Mechanisms with iPathwayGuide. *Curr Protoc Bioinformatics* **57**, 7 15 11-17 15 30 (2017).

11. Cox J, Hein MY, Luber CA, Paron I, Nagaraj N, Mann M. Accurate proteome-wide label-free quantification by delayed normalization and maximal peptide ratio extraction, termed MaxLFQ. *Mol Cell Proteomics* **13**, 2513-2526 (2014).

12. Tyanova S*, et al.* The Perseus computational platform for comprehensive analysis of (prote)omics data. *Nat Methods* **13**, 731-740 (2016).

13. Yuryev A, Kotelnikova E, Daraselia N. Ariadne's ChemEffect and Pathway Studio knowledge base. *Expert Opin Drug Discov* **4**, 1307-1318 (2009).

14. Mallah K*, et al.* Mapping Spatiotemporal Microproteomics Landscape in Experimental Model of Traumatic Brain Injury Unveils a link to Parkinson's Disease. *Mol Cell Proteomics* **18**, 1669-1682 (2019).

15. Heberle H, Meirelles GV, da Silva FR, Telles GP, Minghim R. InteractiVenn: a web-based tool for the analysis of sets through Venn diagrams. *BMC Bioinformatics* **16**, 169 (2015).

16. Pyatnitskiy M, Mazo I, Shkrob M, Schwartz E, Kotelnikova E. Clustering gene expression regulators: new approach to disease subtyping. *PLoS One* **9**, e84955 (2014).

**Supplementary Figures:**

**
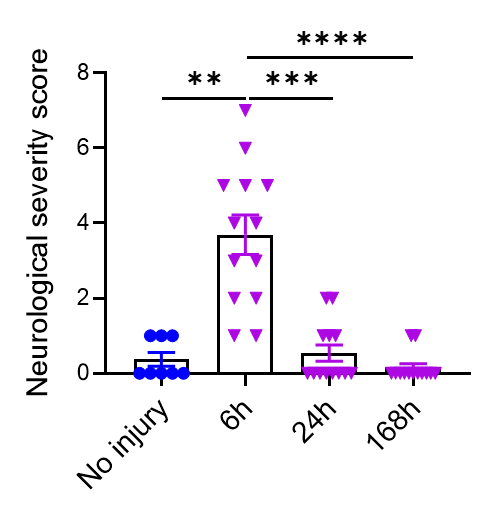
**

**Supplementary Figure 1**: **Neurological Severity Score returns to normal by 24 h post last impact.** The Neurological Severity Score (NSS) score was measured up to 168 h post the 12^th^ injury. Number of replicates: No Injury (n=8), 6h (n=13), 24h (n=13), and 168h (n=13). Statistical analysis was carried out using Kruskal-Wallis test with Dunn’s analysis for multiple comparison of each condition. Median shown, **=p< 0.01, *** =p<0.001, ****=p<0.0001.


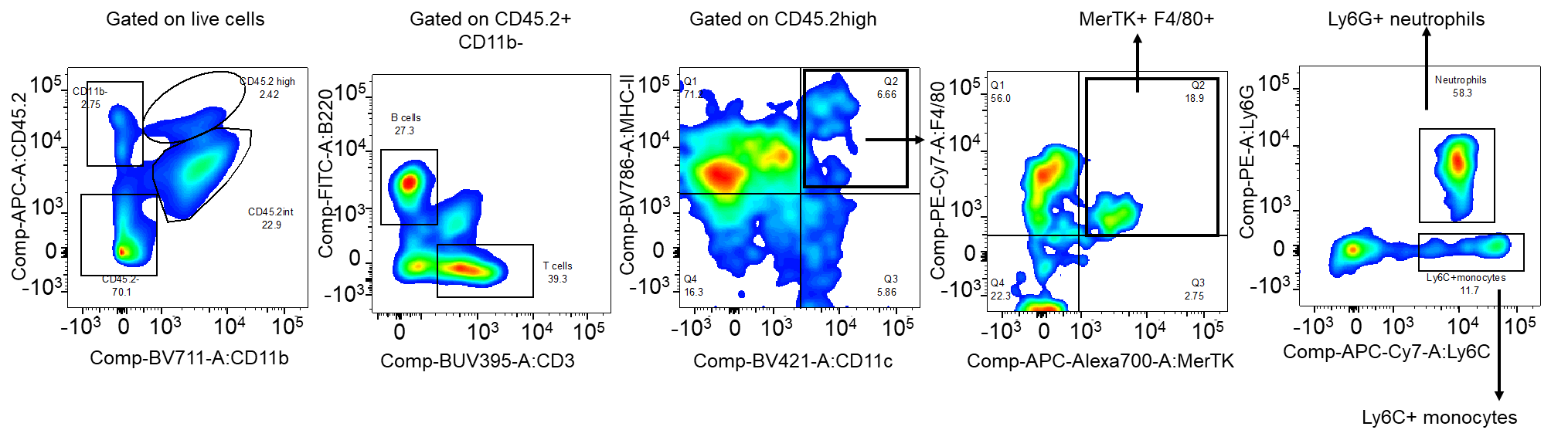


**Supplementary Figure 2**: **Gating strategy for flow cytometry experiments**: The gating strategy for the identification of the different cell populations in the flow cytometry data shown in Figure 1.


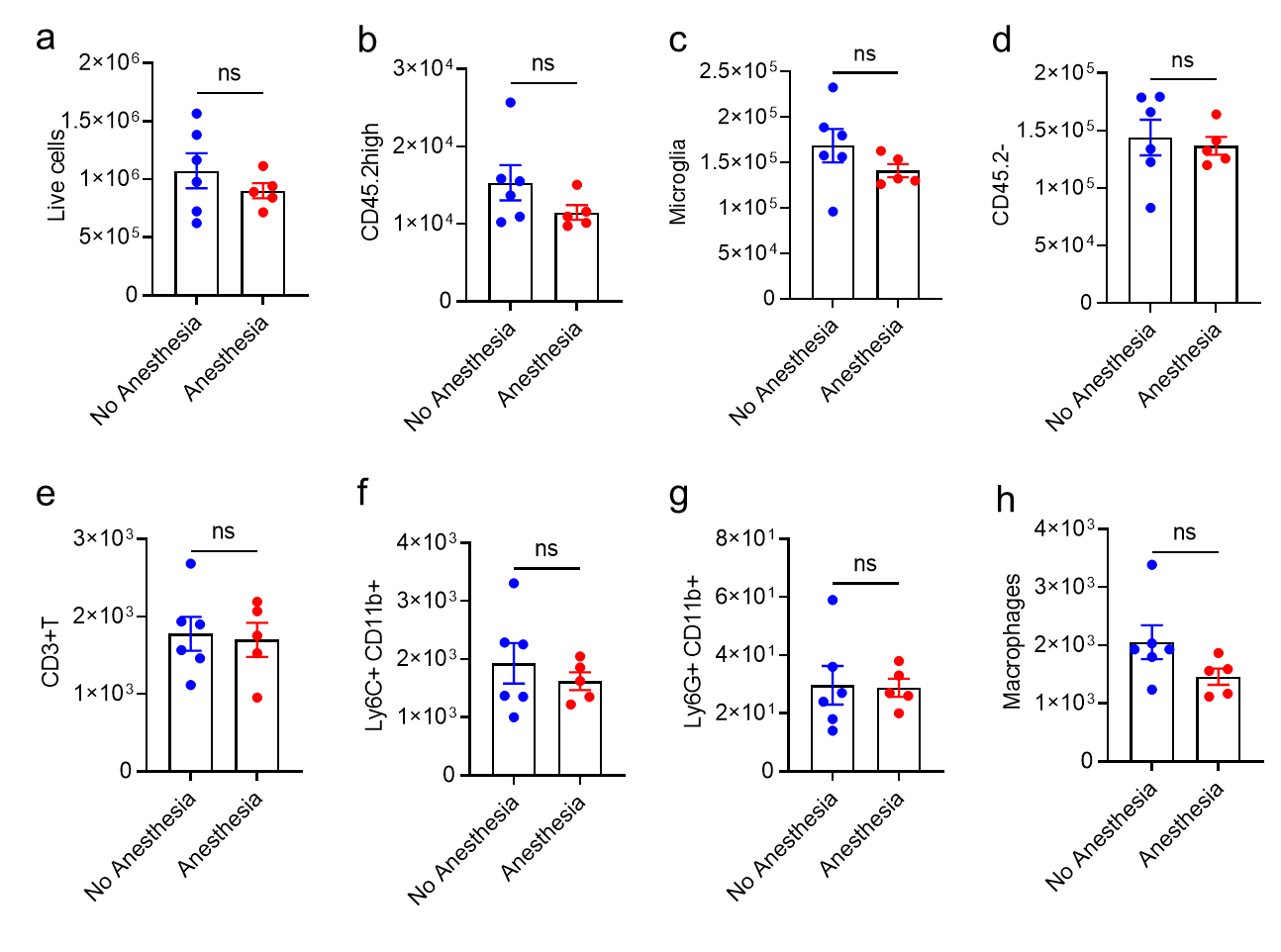


**Supplementary Figure 3: Flow experiments comparing immune and non-immune cells in the brain of no CHI mice without anesthesia and no CHI mice with anesthesia.** Scatter plot showing the total number of cells in the brain of non-injured mice with no anesthesia vs non-injured mice treated with 12 doses of anesthesia. **a)** Total number of live cells. **b)** CD45.2high infiltrating immune cells. **c)** Microglia. **d)** CD45.2- nonimmune cells. **e)** CD3+ T cells. **f)** Monocytes. **g)** Neutrophils and **h)** Macrophages. A minimum of 5 animals/group was used to perform flow cytometry. Statistical analysis was performed using an unpaired t-test. Mean +/- SEM. Ns= no significance.

**
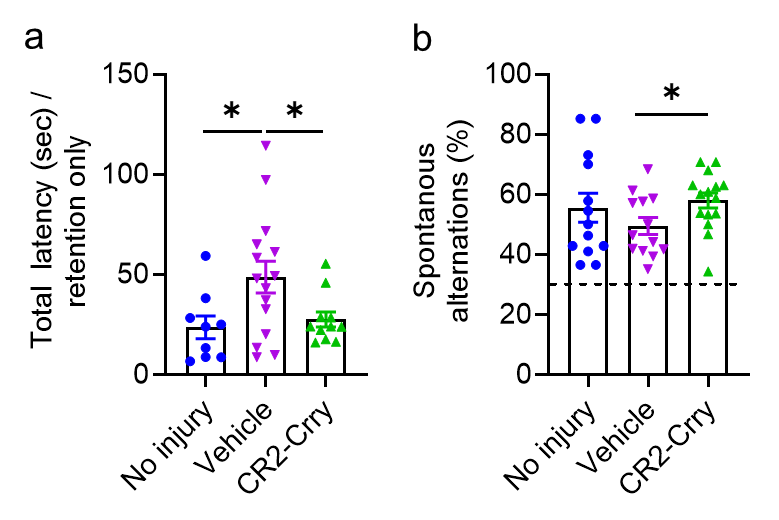
**

**Supplementary Figure 4**: **Cognitive performance is improved with complement inhibition.** **a)** Comparison of memory retention on day 8 of Barnes maze task by measuring total latency to escape the platform. Number of replicates: No Injury (n=9), Vehicle (n=15), CR2Crry (n=11). Statistical analysis was carried out using One-way Anova with Bonferroni post-test for multiple comparisons. An unpaired t-test was also applied for Vehicle vs. CR2Crry. Mean +/- SEM. *p<0.05. **b)** Spontaneous alternations on the Y-maze. Number of replicates: No injury (n=13), Vehicle (n=13), CR2Crry (n=15). Statistical analysis was carried out using One-way Anova with Bonferroni post-test for multiple comparisons. An unpaired t-test was also applied for Vehicle vs. CR2Crry. Mean +/- SEM. *p<0.05.


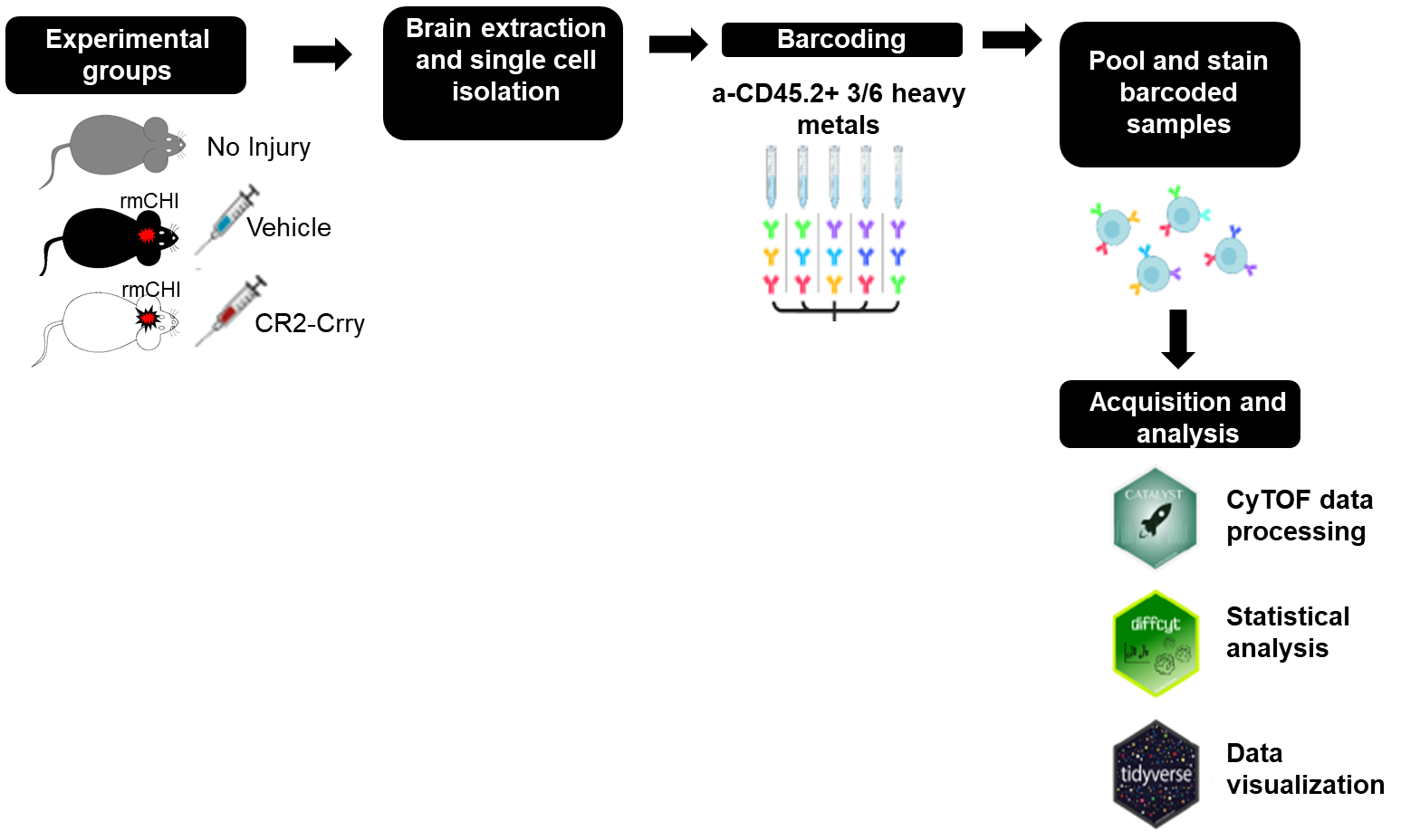


**Supplementary Figure 5**: **Mass Cytometry Workflow.** Graphical workflow of mass cytometry approach used in the study including isolation of the immune cells from the 3 experimental groups, the staining process, and the R packages used to analyze the data.

**
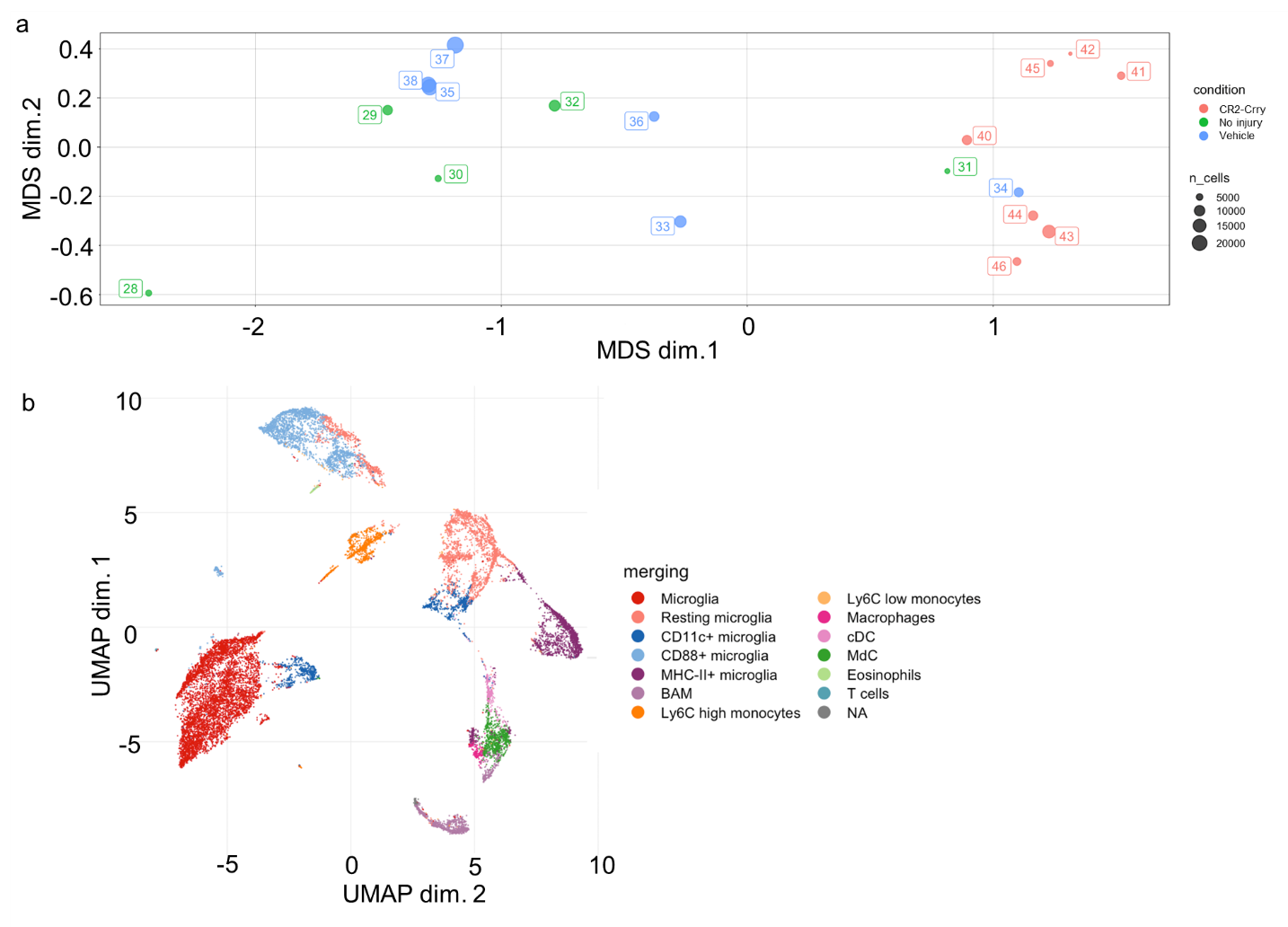
**

**Supplementary Figure 6**: **MDS plot and Lineage UMAP. a)** MDS plot showing the distribution of the samples of the three groups: No Injury (n=5), Vehicle (n=6) and CR2-Crry (7). The circle size is proportional to the number of immune cells in the sample. **b)** A Lineage UMAP plotting the cells based on the expression of the lineage markers. Each color represents a cell population that was identified during the analysis. 1000 cells per sample were used and 18,000 cells are shown in the UMAP for display.

**
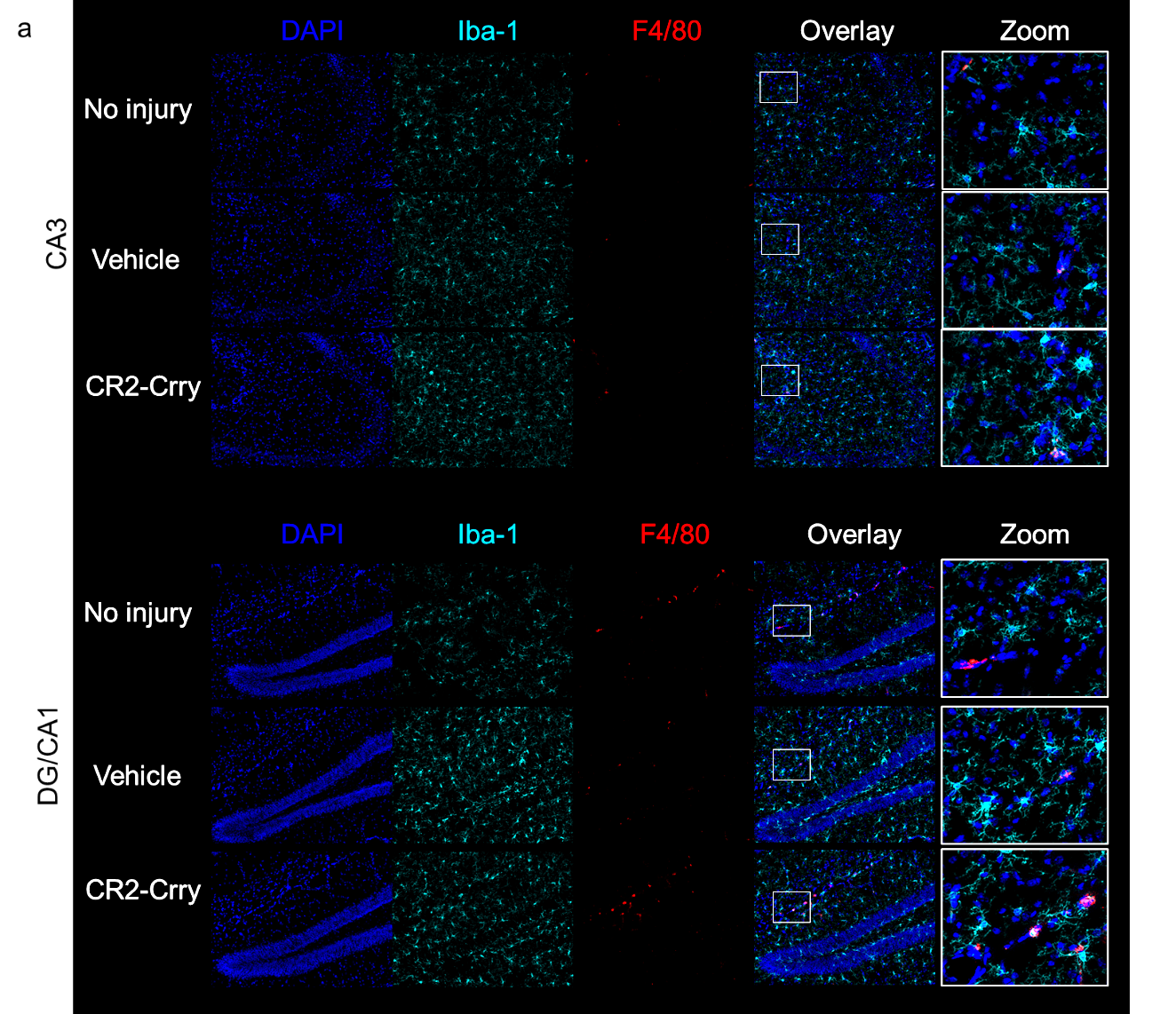
**

**
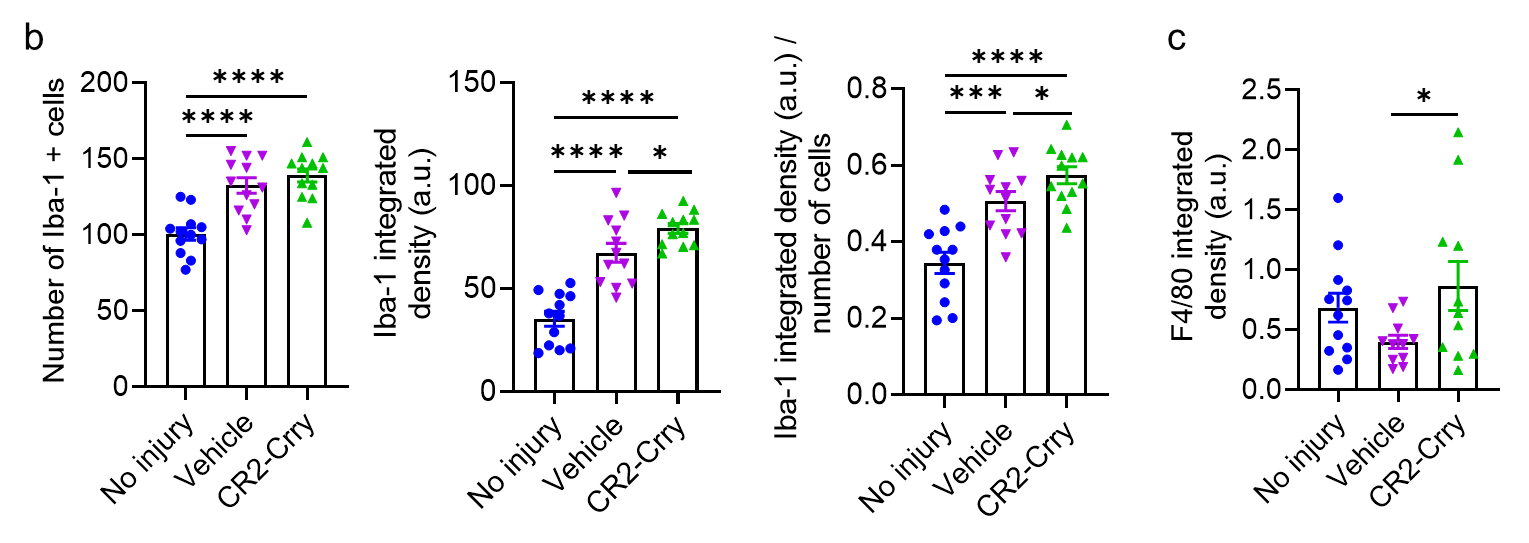
**

**Supplementary Figure 7:** **IF staining of Iba-1 and F4/80. a)** 20x Immunofluorescence staining for Iba 1 (cyan) and F4/80 (red) in the CA1 and DG/CA3 regions from brains collected at 21 days after the 12-impact in vehicle and CR2-Crry conditions as well as non-injured control. **b)** Quantification of Iba-1 staining for the following parameters: number of cells, integrated density, and integrated density/number of cells. **c)** Quantification of F4/80 staining. For B and C, Statistical analysis was carried out using One-way Anova with Bonferroni post-test for multiple comparisons. Also performed was an unpaired t-test between vehicle and CR2-Crry mice. Mean +/- SEM. *= p<0.05; ***= p<0.001; ****= p<0.0001. For B and C, three animals per condition (i.e. no injury, vehicle, and CR2-Crry) were used and four images per animal were acquired.


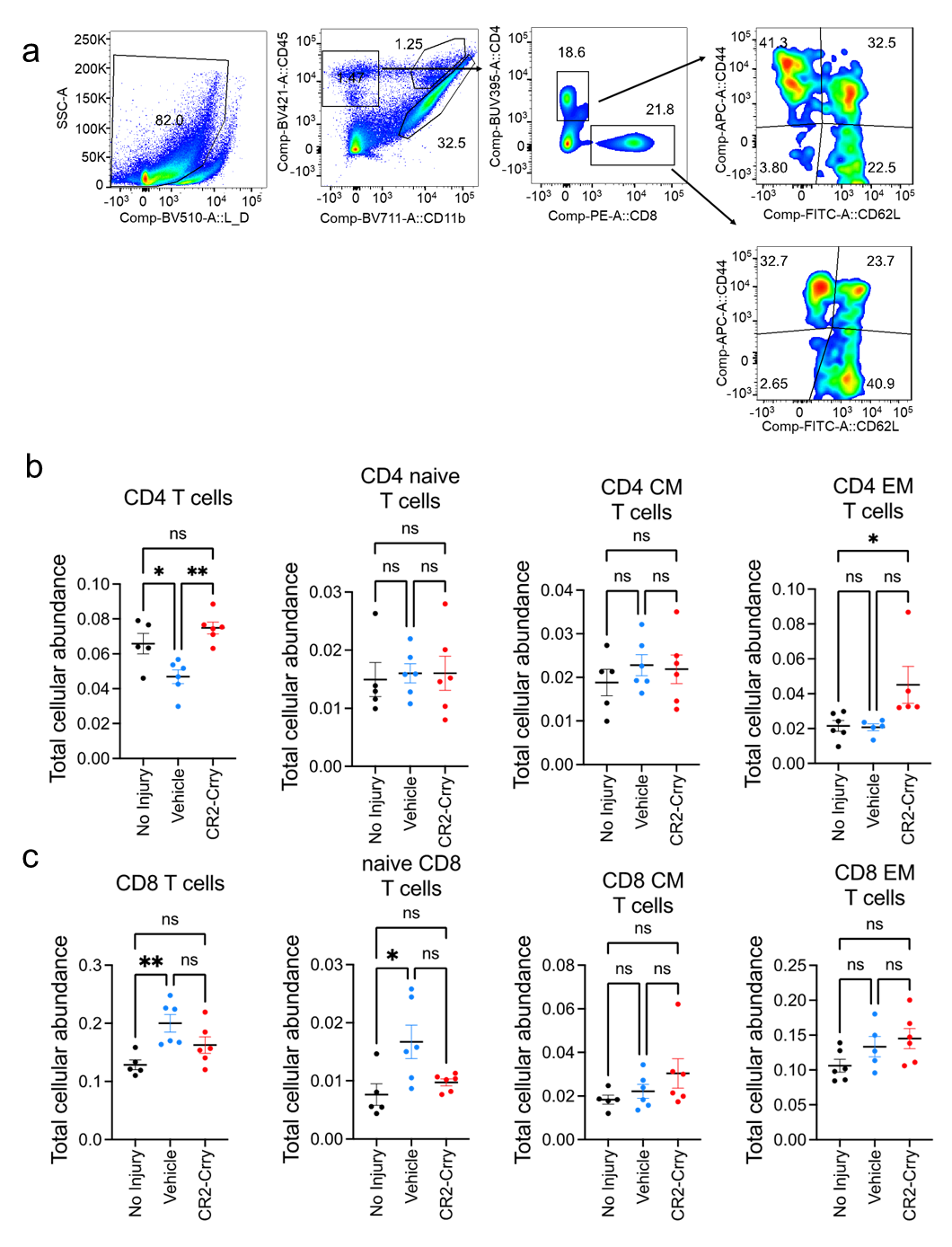


**Supplementary Figure 8. T cell subsets in the brain.** Single cell suspensions from the brain of non-injured, vehicle and CR2-Crry treated mice were analyzed by flow cytometry and naïve (N), central memory (CM) and effector memory (EM) T cells subsets for CD4 (B) and CD8 (C) were identified using CD62L and CD44 marker expression as detailed in the gating strategy shown in panel A). N=6 animals/group. Significance was calculated using one way Anova with Bonferroni post-test (ns=not significant, *p<0.05; ** p<0.01).


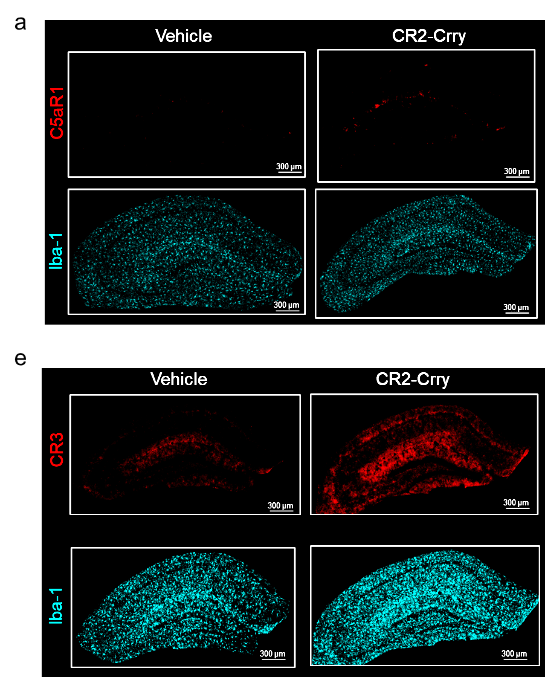

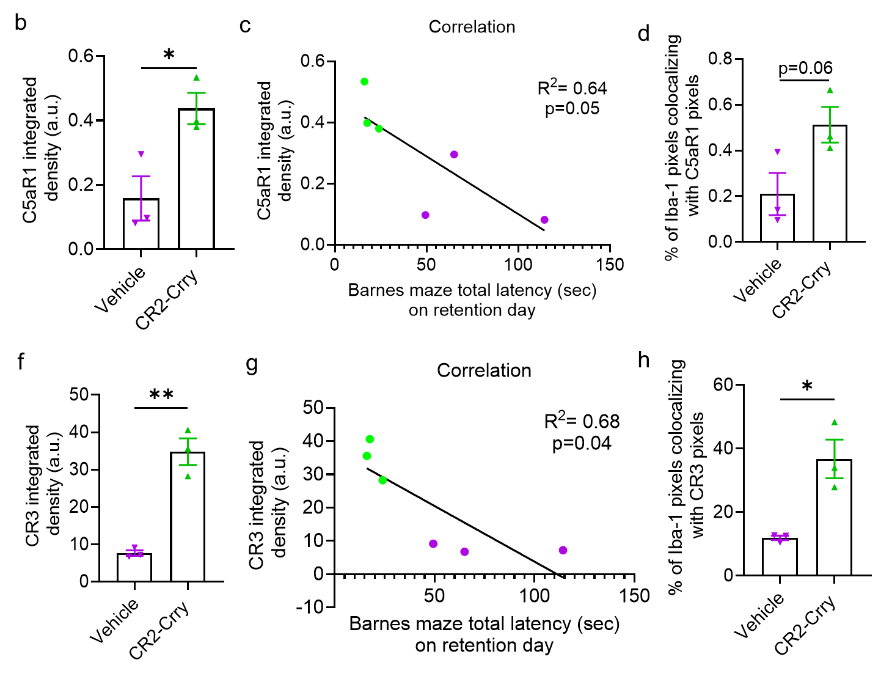


**Supplementary Figure 9:** **IF staining of C5aR1, CR3, and Iba-1. a)** 20x stitched immunofluorescence stained image of C5aR1 (red) and Iba-1 (cyan) in the hippocampus of brains collected from mice sacrificed at 21 days after the 12^th^ impact in vehicle and CR2-Crry conditions. **b)** Quantification of C5aR1 integrated density across all three conditions. **c)** Correlation between the C5aR1 integrated density and the Barnes Maze total latency on the retention day. Shown on the graph are the R^2^, P-value, and N for Pearson’s coefficient for linear correlation. Green dots: CR2-Crry; Purple dots: Vehicle. **d**) The % of Iba-1+ pixels that are colocalizing with C5aR1 pixels. This was computed by dividing the number of colocalized pixels of C5aR1/Iba-1 by the total number of Iba-1+ pixels. For B and D, statistical analysis was carried out using an unpaired t-test. Mean +/- SEM. *= p<0.05. The number of replicates= three animals per condition. Each data point represents the average value of the measured variable within the hippocampus from both brain hemispheres of the same animal**. e)** 20x stitched immunofluorescence stained image of CR3 (red) and Iba-1 (cyan) in the hippocampus of brains collected from mice sacrificed at 21 days after the 12^th^ impact in vehicle and CR2-Crry conditions. **f)** Quantification of CR3 integrated density across all three conditions. **g)** Correlation between the CR3 integrated density and the Barnes Maze total latency on the retention day. Shown on the graph are the R^2^, P-value, and N for Pearson’s coefficient for linear correlation. Green dots: CR2-Crry; Purple dots: Vehicle. **h)** The % of Iba-1+ pixels that are colocalizing with CR3 pixels. This was computed by dividing the number of colocalized pixels of CR3/Iba-1 by the total number of Iba-1+ pixels. For F and H, statistical analysis was carried out using an unpaired t-test. Mean +/- SEM. *= p<0.05;**= p<0.01. The number of replicates=3 animals per condition. Each data point represents the average value of the measured variable within the hippocampus from both brain hemispheres of the same animal.


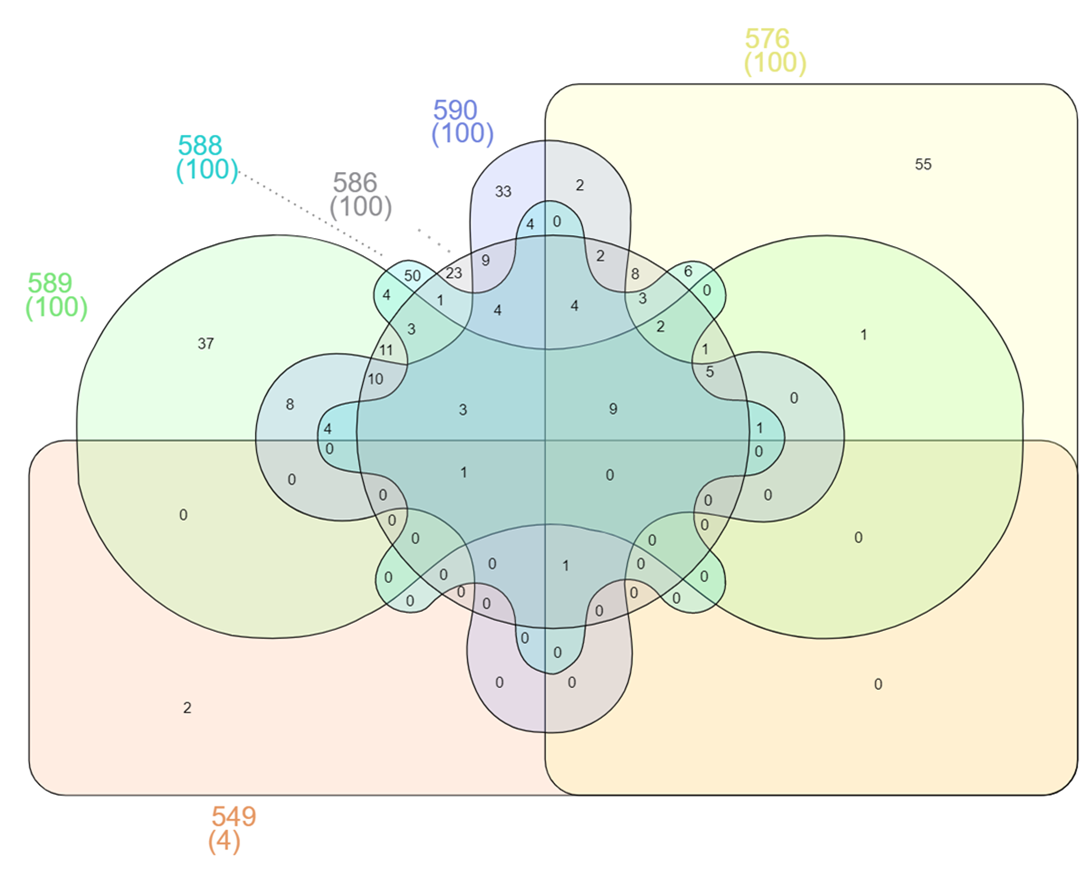


**Supplementary Figure 10: Venn diagram of Protein Pathways**. Venn diagram of the unique and common pathways associated with the proteins of each of the 6 identified clusters obtained from the proteomics heatmap in Figure 6a.

**Supplementary Table 1:** List of antibodies used in the mass cytometry experiments.

| **Marker** | **Company** | **Clone** | **Metal** | **Isotope** |
| --- | --- | --- | --- | --- |
| **CD11b** | Biolegend | M1/70 | Nd | 148 |
| **Ly6C** | Biolegend | HK1.4 | Nd | 150 |
| **MHC-II** | Biolegend | M5/114.15.2 | Yb | 174 |
| **C5aR1** | Biolegend | 20/70 | Gd | 160 |
| **CD16/32** | Biolegend | 93 | Er | 167 |
| **F4/80** | Biolegend | BM8 | Tb | 159 |
| **CD64** | Biolegend | X54-5/7.1 | Eu | 151 |
| **CX3CR1** | Biolegend | SA011F11 | Dy | 164 |
| **P2Y12R** | Biolegend | S16007D | Dy | 162 |
| **CD172** | Biolegend | P87 | Yb | 173 |
| **CD38** | StandardBiotools | 90 | Lu | 175 |
| **CD206** | Biolegend | C068C2 | Er | 168 |
| **CD163** | Biolegend | S15049I | Er | 170 |
| **CD14** | Biolegend | Sa14-2 | Gd | 156 |
| **CD86** | StandardBiotools | GL-1 | Yb | 172 |
| **MerTK** | Biolegend | 2B100C42 | Nd | 143 |
| **P2X7R** | Biolegend | 1F11 | Nd | 142 |
| **Ly6G** | Biolegend | 1A8 | Pr | 141 |
| **B220** | Biolegend | RA3-6B2 | Nd | 144 |
| **CD36** | Biolegend | HM36 | Nd | 146 |
| **Ter119** | StandardBiotools | TER-119 | Sm | 154 |
| **SiglecF** | BD | S17007L | Nd | 145 |
| **CCR2** | R&D | MAB55381 | Dy | 163 |
| **C3aR** | Hycult | 14D4 | Dy | 161 |
| **CD117** | Biolegend | 2B8 | Er | 166 |
| **CD45** | Biolegend | 30-F11 | Sm | 147 |
| **SiglecH** | Biolegend | 551 | Gd | 158 |
| **CD3** | Biolegend | 145-2C11 | Sm | 152 |
| **NK1.1** | Biolegend | PK136 | Ho | 165 |
| **PD-L1** | BioXcell | 10F.9.G2 | Eu | 153 |
| **FcER1a** | StandardBiotools | 03-01 | Yb | 176 |
| **CD44** | StandardBiotools | IM7 | Yb | 171 |
| **CD11c** | Biolegend | N418 | Bi | 209 |
| **Sca-1** | StandardBiotools | D7 | Tm | 169 |

**Captions for Supplementary Datasets 1 to 3:**

**Supplementary Dataset 1:** List of proteins identified in the 6 clusters.

**Supplementary Dataset 2:** List of the top 100 pathways identified in each of the 6 clusters.

**Supplementary Dataset 3:** Lists of common and unique pathways of each of the 6 clusters.

**Captions for Supplementary Movies 1 to 4:**

**Supplementary Movie 1:** 3D video of microglia image of the No Injury condition (position 2) of Figure 2b showing all microglia within the image.

**Supplementary Movie 2:** 3D video microglia image of the No Injury condition (position 2) of Figure 2b showing the single zoomed-in microglia.

**Supplementary Movie 3:** 3D video of microglia image of the 12-impacts condition (position 2) of Figure 2b showing all microglia within the image.

**Supplementary Movie 4:** 3D video microglia image of the 12-impacts condition (position 2) of Figure 2b showing the single zoomed-in microglia.
